# Mapping differential responses to cognitive training using machine learning

**DOI:** 10.1111/desc.12868

**Published:** 2019-07-22

**Authors:** Joseph P. Rennie, Mengya Zhang, Erin Hawkins, Joe Bathelt, Duncan E. Astle

**Affiliations:** ^1^ MRC Cognition and Brain Sciences Unit University of Cambridge Cambridge UK

**Keywords:** cognitive training, development, individual difference, machine learning

## Abstract

We used two simple unsupervised machine learning techniques to identify differential trajectories of change in children who undergo intensive working memory (WM) training. We used self‐organizing maps (SOMs)—a type of simple artificial neural network—to represent multivariate cognitive training data, and then tested whether the way tasks are represented changed as a result of training. The patterns of change we observed in the SOM weight matrices implied that the processes drawn upon to perform WM tasks changed following training. This was then combined with *K*‐means clustering to identify distinct groups of children who respond to the training in different ways. Firstly, the *K*‐means clustering was applied to an independent large sample (*N* = 616, *M*_age_ = 9.16 years, range = 5.16–17.91 years) to identify subgroups. We then allocated children who had been through cognitive training (*N* = 179, *M*_age_ = 9.00 years, range = 7.08–11.50 years) to these same four subgroups, both before and after their training. In doing so, we were able to map their improvement trajectories. Scores on a separate measure of fluid intelligence were predictive of a child's improvement trajectory. This paper provides an alternative approach to analysing cognitive training data that go beyond considering changes in individual tasks. This proof‐of‐principle demonstrates a potentially powerful way of distinguishing task‐specific from domain‐general changes following training and of establishing different profiles of response to training.

## Graphical abstract

(a) Pre‐ and post‐ working memory (WM) training datasets from 179 children were used to train self‐organizing maps (SOM), a unsupervised machine learning algorithm that represents multivariate task relationships in a 2‐dimentional space, (b) WM task relationships as represented by SOM changed as a result of training, indicating that the processes drawn upon to perform these tasks were altered. Improvements might be due to task‐specific rather than domaingeneral enhancement and (c) There were differential improvement profiles among children and and independent measure of fluid intelligence at pre‐traini

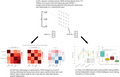

ng is predictive of these profiles.

**Research Highlights**

- We used a multivariate approach to understand cognitive training mechanisms—unsupervised machine learning.
- Following training, task relationships change, implying that the cognitive processes drawn upon to perform these tasks have changed.
- The learning algorithm also learnt that there were differential improvement trajectories among children and an independent measure of fluid intelligence is predictive of these trajectories.

## 1 INTRODUCTION

Working memory (WM), the ability to hold and manipulate information in the mind for brief periods of time, is predictive of healthy cognition across the lifespan and closely linked to academic attainment, employability and well‐being (Diamond, 2012). Consequently, the prospect of enhancing WM and closely associated cognitive skills such as attention, processing speed and reasoning via cognitive training has received considerable interest from researchers and commercial enterprises (Diamond, 2012; Green & Bavelier, 2008; Hertzog, Kramer, Wilson, & Lindenberger, 2008). The assumption being that enhancing this general‐purpose system will produce wide benefits to other aspects of cognition and learning.

Cognitive training studies typically use a range of assessment tasks to test the effect of training. These are delivered before and after extended practice on a different set of training tasks. Studies designed to test whether the training is effective typically compare these training effects against an active control condition and correct for multiple comparisons across assessments (Simons et al., 2016). Thus far, evidence for improvements on tasks similar to those practised is plentiful. In contrast, evidence for improvements on more distant tasks is limited (Gathercole, Dunning, Holmes, & Norris, 2019; Green & Bavelier, 2008; Hertzog et al., 2008; Melby‐Lervag, Redick, & Hulme, 2016; Melby‐Lervag & Hulme, 2016). Indeed, the transferability of training is substantially modulated by the degree of overlap between the training activities and the untrained assessment tasks (Gathercole et al., 2019).

The role of individual differences in the size of training effects is receiving increasing attention from researchers. The longest‐standing example of this is the aptitude by the treatment interaction (e.g., Cronbach, 1957; Ferguson, 1956; Snow, 1989), or in other words, how an individual's current cognitive ability interacts with their training outcome. Two popular accounts have emerged, namely: the compensation account and the magnification account (Lövdén, Brehmer, Li, & Lindenberger, 2012). The compensation account suggests that those with higher baseline scores have less to gain, being closer to ceiling prior to training. This assumes that there is plateau in overall performance, with some subjects being closer to this before they start training. Conversely, the magnification account suggests that those with higher baseline scores will show greater improvements, because they have more cognitive resources available in order to maximize on the potential benefit of the training—for example to develop strategies (see Karbach, Könen, & Spengler, 2017, for a recent overview). These extreme accounts are likely oversimplifications (Smoleń, Jastrzebski, Estrada, & Chuderski, 2018). Noetheless, understanding prior factors that predict transfer effects may help explain the many inconsistencies concerning the effectiveness of cognitive training; it could also help tailor training towards those most responsive. Thus far, studies examining individual differences in training are relatively rare, but are steadily growing in number. The majority have explored the impact of known pre‐training individual differences, such as age (Borella et al., 2014; Schmiedek, Lövdén, & Lindenberger, 2010), baseline cognitive performance (Bürki, Ludwig, Chicherio, & Ribaupierre, 2014; Guye, Simoni, & Bastian, 2017; Zinke et al., 2014) and cognition‐related beliefs (e.g., malleability of intelligence; Jaeggi, Buschkuehl, Shah, & Jonides, 2014). They provide evidence that some pre‐training individual differences may explain variability in training effects.

The majority of these studies has used univariate analytical techniques (e.g., Jaeggi, Buschkuehl, Jonides, & Perrig, 2008; Zinke et al., 2014). That is, taking single tasks and testing whether performance on them changes significantly following training, and whether this is moderated by a known individual difference factor. A principal challenge to this approach is task impurity—the extent to which any given task measures an intended construct—because this makes it difficult to identify what mechanism is being trained (Burgess, 2004; Hasson, Chen, & Honey, 2015; Meyer et al., 2001; Miyake et al., 2000). For example, both N‐back and complex span tasks purportedly measure “WM capacity,” but training effects on these tasks do not consistently transfer to one another (Harrison et al., 2013; Li et al., 2008). Similarly, both letter span and word span tasks purportedly measure “verbal short‐term memory,” but training effects on letter span do not always transfer to word span (Ericcson, Chase, & Faloon, 1980). In short, the labels assigned to tasks do not always correspond well to the underlying processes taxed by the assessment, or those enhanced via practice. Comparing individual tasks before and after training does not overcome this challenge, because changes on individual measures could stem from changes in multiple different underlying processes (Protzko, 2017). As a result, a number of researchers are now beginning to explore the potential value of multivariate approaches to considering changes that occur following cognitive training.

One such approach is structural equation modelling (SEM), in which cognitive abilities are represented by latent constructs (Karbach et al., 2017; Schmiedek et al., 2010). Schmiedek and colleagues conducted a large training study, in which they used Latent Score Change Modelling (a form of SEM) and found transfer effects to be detectable at a latent level. As they note, it is possible to observe significant changes at the latent level despite non‐significant changes at a task‐specific level and vice versa. This is presumably because latent constructs may change substantially, but their contribution to any single task in the battery could be relatively small. Conversely, we might observe highly specific practice effects particular to a given paradigm or stimulus set (e.g., letters or digits) that do not stem from changes to any broader underlying latent construct. This can also be a powerful tool for looking at individual differences because it accounts for measurement error in observed variables and thus provides a good way of establishing stable individual differences (Hamaker, Kuiper, & Grasman, 2015). This has enabled some researchers to investigate individual differences by including separate predictors for the estimated change variable in their models (e.g., Bürki et al., 2014; Guye et al., 2017; Karbach et al., 2017; Lövdén et al., 2012).

Although promising, this method is not without its drawbacks. Confirmatory factor analysis approaches require researchers to make subjective choices (albeit based on theory) about the structure of underlying components from the many possible configurations at differing levels of granularity. Furthermore, establishing training effects is particularly challenging because the nature of the underlying constructs, their interrelationships or their task loadings may have changed substantially as a function of training. Investigators are faced with a dilemma: they could fit the same model both before and after training, allowing for a meaningful comparison of model parameters but ignoring the fact that this model may no longer be the most appropriate. Alternatively, they could fit the best model separately before and after training, which would allow for the best representation of the underlying components, but render direct comparisons less meaningful.

Machine learning provides an alternative to modelling task relationships. Unsupervised learning algorithms hold the same advantage as other data‐driven methods such as principal component analysis (PCA) and exploratory factor analysis (EFA), in that they allow researchers to explore task relationships without requiring subjective judgements to be made about their nature a priori. Machine learning algorithms also lend themselves well to non‐linearities in multidimensional data, allowing them to capture more nuanced task relationships compared with commonly used linear methods (general linear regression, factor analysis, PCA etc.). Some algorithms cluster participants in a competitive manner, rather than clustering tasks at the whole‐group level (as would be the case for PCA or EFA). These may be particularly useful when we suspect there could be large individual differences—in the context of training,resulting in differing profiles of change following an intervention. Iterative clustering techniques can provide a data‐driven way of subgrouping participants and thereby reveal different profiles of performance. This has the potential to enable researchers to explore individual differences in training in a different way—rather than testing whether gains in training are predicted by known factors (e.g., age, baseline ability); it might allow researchers to identify individual differences in the *profile of the training response itself*. Despite these potential benefits, we know of no attempts to use machine learning to understand transfer effects following cognitive training. This paper aims to explore the utility of combining two relatively simple machine learning techniques, namely: self‐organizing maps (SOMs) and *K*‐means clustering, to explore task relationships and how these might be altered by training in two large datasets.

First proposed by Kohonen (1990), SOMs belong to a family of artificial neural networks and provide a way of organizing multidimensional data into a lower dimensional space, represented as a topographical distribution. An unsupervised learning algorithm projects the original data from a multidimensional input space onto a two‐dimensional grid of nodes called a map. Each node corresponds to a node‐weight vector with the same dimensionality as the number of input variables, thereby producing an inter‐variable representational space, wherein the geometric distance between nodes corresponds to the degree of similarity in the input data associated with them (Kohonen, 2014). This enables key inter‐variable relationships existing in multidimensional space to be identified and accentuated. Moreover, this allows the researcher to explore the overlap in representational space between tasks and how, or whether, this changes as a result of the training. Once established, SOMs can be used to generate quantitative predictions about training effects in unseen data, something currently underutilized in cognitive training research.

Subsequently, a *K*‐means clustering algorithm can be used to identify relatively homogenous subgroups (i.e., “clusters”) within the multidimensional node‐weight vector space produced by the SOM algorithm. This allows for the exploration of individual differences in task relationships and makes use of information that would otherwise be lost. Identifying data‐driven subgroups with distinct cognitive profiles could be a valuable way of understanding different trajectories in cognitive change. We wanted to establish a method for doing this.

## 2 METHODS

This section contains a brief description of the SOM algorithm and its generic implementation, followed by a stepwise account of the analyses performed on two datasets containing the same set of tasks.

### 2.1 SOM algorithm

**Figure 1:**
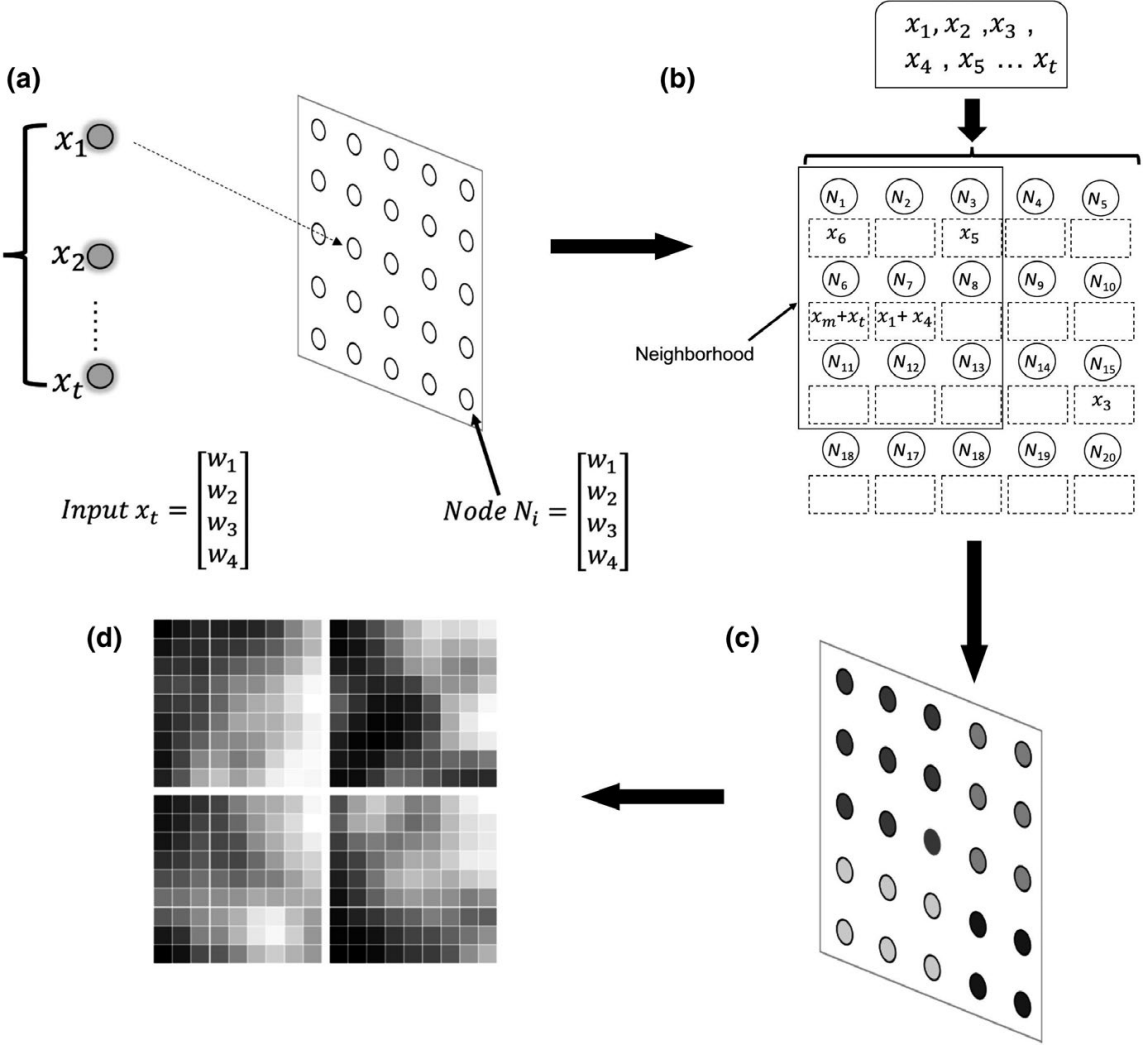
Illustration of SOM batch training steps to update node weights using given dataset. (a) Each input vector ***x***(***t***) is mapped onto its Best Matching Unit. (b) All input vectors in each node are summed and used to update its Best Matching Unit and neighbourhood, which shrinks with time. (c) When training completes, SOM has preserved the topological information of the input data. Data with similar inter‐variable relationships are assigned to closer Best Matching Units. (d) Visualization of individual node weights (*w_n_*), namely the component planes, when input vector contains four variables. Abbreviation: SOM, self‐organizing map

SOMs were trained using the neural network toolbox in (MATLAB and Statistics Toolbox Release). SOMs consist of a predefined number of nodes laid out on a two‐dimensional grid plane. Each node corresponds to a weight vector with the same dimensionality as the input data. We initialized the node‐weight vectors using linear combinations of the first two principal components of the input data. SOMs were then trained using a batch implementation (see Figure 1 for a graphical overview), in which each node ***i*** is associated with a model ***m_i_*** and a “buffer memory.” One cycle of the batch algorithm can be broken down into the following: Each input vector, in this case a single child's performance profile across the four assessment tasks, ***x***(***t***) is mapped onto the node with which it shares the least Euclidean distance at time ***t***. This node is known as its Best Matching Unit. Each buffer sums the values of all input vectors ***x***(***t***) in the neighbourhood set belonging to node ***i*** and divides this by the total number of these input vectors to derive a mean value. All ***m_i_*** are then updated concurrently according to these values. In this way, neighbouring nodes become more similar to one another. This cycle is repeated, clearing all the buffers on each cycle and distributing new copies of the input vectors into them. The neighbourhood size (***ND***) decreases as a function of ***t*** over ***n*** steps in an “ordering” phase, from the initial neighbourhood size (***INS***) down to 1 (Equation 1). In the “fine‐tuning” phase the neighbourhood size is fixed at <1, meaning that the node weights are updated according only to the input vectors for which they are the Best Matching Unit. This node adjustment process is the mechanism by which the SOM learns about the input data.

(1) $$\mathrm{ND}=1+\left[\left(\mathrm{INS}\right)\times \left(1-\left(\frac{t}{n}\right)\right)\right]$$

### 2.2 Cognitive assessments

Four span tasks from the automated working memory assessment battery (AWMA; Alloway, 2007) were used in the current analysis. In *Forward Digit Recall*, participants hear a sequence of numbers and are required to repeat them back out loud, in the same order in which they were presented; *Backward Digit Recall*, participants hear a sequence of numbers and are required to repeat them back out loud, in the reverse of the presentation order. These tasks purport to measure verbal short‐term and WM respectively. *Dot Matrix*, participants see a sequence of dots in a 3 × 3 matrix and are required to recall the order and position of the dots by pointing to a blank 3 × 3 response matrix; *Mr.X*, participants are present with sequences of two cartoon characters placed next to one another, both of which are holding a ball in one of their two outstretched arms, and the one on the right is rotated to varying degrees on each presentation. For each pair of Mr. X's participants are required to make a same‐different judgement with regard to whether they are holding the ball in the same hand, whilst retaining the spatial information as to where the ball held by the right‐hand Mr. X resides. They are then required to recall the previously retained spatial locations in the correct order by pointing to one of the eight locations represented by dots in a circle. These tasks purport to measure visuospatial short‐term and WM respectively. All tasks along with the instructions are computerized and practice trials were completed on each to help ensure comprehension.

### 2.3 Participants

We used three relatively large datasets in this analysis. All datasets consisted of age‐standardized data (i.e., *M* = 100, *SD* = 15) from the four AWMA tasks. In the following sections, we describe the datasets we used, and summary scores are described in Table 1.

**Table 1:** Summary statistics of task performance in the respective datasets

Dataset			Forward Digit	Dot Matrix	Backward Digit span	Mr. X
CALM (*N = *526)		*M*	91.93	91.55	90.80	97.45
		*SD*	15.98	15.17	13.44	14.86
ACE (*N = *90)		*M*	104.14	103.71	103.65	105.58
		*SD*	13.38	14.97	14.77	15.60
CALM + ACE (*N = *616)		*M*	93.86	93.46	92.81	98.64
		*SD*	15.84	15.41	14.00	15.26
Pre‐training	Adaptive (*N = *179)	*M*	93.95	90.78	85.58	89.73
		*SD*	15.58	16.12	14.90	16.16
	Non‐adaptive (*N* = 70)	*M*	90.93	94.29	84.84	91.34
		*SD*	16.27	16.93	13.04	15.88
Post‐training	Adaptive (*N = *179)	*M*	100.54	110.56	101.01	102.53
		*SD*	17.64	19.59	15.43	18.68
	Non‐adaptive (*N* = 70)	*M*	91.94	103.51	96.32	100.83
		*SD*	18.01	18.89	17.23	20.91

Note

The expected mean and standard deviation of the normative AWMA data is 100.00 and 15.00 respectively.

Abbreviations: ACE, Attention and Cognition in Education; AWMA, automated working memory assessment; CALM, Centre for Attention, Learning and Memory.

#### 2.3.1 Centre for Attention, Learning and Memory

The first dataset comprised of data collected from 526 participants (*M* = 9.16 years, range: 5.16–17.91 years, *SD* = 2.16 years; 171 girls) who had completed assessments as part of the Centre for Attention, Learning and Memory (CALM) initiative. This is a study of children referred based on ongoing problems in attention, learning and memory. Children visit the MRC Cognition and Brain Sciences Unit and undergo a wide battery of cognitive and behavioural assessments, which includes the four tasks described above.

#### 2.3.2 Attention and Cognition in Education

This sample was collected for a study investigating the neural, cognitive and environmental markers of risk and resilience in children. Ninety typically developing children who attend mainstream schools in the UK (*M = *9.42 years, range = 6.91–12.58 years, *SD* = 1.49 years; 45 girls) and their families were invited to the MRC Cognition and Brain Sciences Unit in Cambridge for a comprehensive cognitive assessment, which included the four tasks described above.

In later analyses, we combined data from the two above‐mentioned studies for better statistical power and larger individual variability in task profiles, which is desirable for a “baseline” dataset.

#### 2.3.3 Combined training studies

This dataset comprised of pre‐training and post‐training data collected from 179 participants (*M* = 9.00 years, range 7.08–11.50 years, *SD* = 1.06 years; 45 girls, combined over several independent training studies (Dunning, Holmes, & Gathercole, 2013; Holmes, Gathercole, & Dunning, 2009; Holmes et al., 2010; Holmes et al., 2015). Inclusion criteria varied across studies, such as low WM score on standard tests (Dunning et al., 2013; Holmes et al., 2009), low language abilities (Holmes et al., 2015) or Attention Deficit Hyperactivity Disorder (ADHD) diagnosis (Holmes et al., 2010). All children participated in the standard Cogmed RM program (see Klingberg et al., 2005, for a detailed description of the training tasks), which involved 20–25 sessions of adaptive training on temporary storage and manipulation of sequential visuospatial or verbal information, or both. Detailed methods regarding training program have been reported previously. The same four AWMA tasks were administered before and after training as measures of WM transfer, leading to a total of eight variables for this dataset. Participants showed significant improvement on all four tasks in the post‐training assessment (*p* < 0.001) compared to their baseline assessments, most notably on Dot Matrix and Backwards Digit Span (Cohen's *d*: Forward Digit = 0.395, Dot Matrix = 1.103, Backward Digit = 1.017, Mr. X = 0.732). We also included summaries for a combined control group who were given a non‐adaptive version of the Cogmed training (*M = *9.02 years, range = 7.50–10.50 years, *SD* = 0.72 years; 29 girls). Corresponding analysis of variance (ANOVA) established significant treatment by time interactions for Forward Digit, *F*(1, 69) = 4.58, *p* < 0.05; Dot Matrix, *F*(1, 69) = 27.36, *p* < 0.001; Backward Digit, *F*(1, 69) = 9.62, *p* < 0.01; Mr. X, *F*(1, 69) = 4.59, *p* < 0.05. In all cases, the improvements were significantly greater for the training group than the control group. Furthermore, simple main effect analysis showed that the performances of control group on all tasks were significantly better at post‐training than pre‐training (*p* < 0.001), except for Forward Digit (*p* = 0.48).

### 2.4 Analysis pipeline

#### 2.4.1 Training SOMs

The SOM learning algorithm and model require the selection of several parameters, including the number of map nodes, initial neighbourhood size, the ordering phase length, and fine‐tuning phase length. These hold important theoretical, computational and statistical implications. However, according to Kohonen (2014), there are no standard mathematical definitions to inform the selection of such parameters. Instead, Kohonen covers some key concepts and provides suggestions based on experience. A detailed discussion of this topic is beyond the scope of this paper. However, for a more detailed explanation of our selection process and an overview of the results, see the Supporting Information provided on this topic. In short, we selected parameters with the aim that the SOM model would represent the training sample well, whilst still maintaining generalizability to the wider population. We trained the following three SOMs: (a) a SOM trained on the combined CALM and Attention and Cognition in Education (ACE) dataset (CALM/ACE); (b) a SOM trained on the pre‐training dataset; and (c) a SOM trained on the post‐training dataset. The relatively large sample size of CALM/ACE (616 participants) provided a good baseline dataset for learning about the overlap between the different tasks, and the possible cognitive profiles that exist. The smaller training datasets were used to investigate questions about training effects.

#### 2.4.2 Does the SOM model represent the samples well?

##### Cross‐validation

The first step after fitting a model is to test its validity. We applied a cross‐validation procedure to test the null hypothesis that the SOM does not estimate unseen data above chance levels. Specifically, this involved randomly removing 20% of the CALM/ACE data (i.e., approximately 120 participants), then using the remaining 80% to fit a SOM, which was used to predict the reserved data. The prediction was made with a technique called K‐Nearest Neighbours (Altman, 1992), in which the value of the to‐be‐predicted variable is decided by the values of the three closest SOM nodes in terms of Euclidian distance with respect to the vector containing the other‐unseen variables. For example, if Forward Digit is the target variable, a subject's scores on the other three tasks will be fed to the algorithm to find the three nearest SOM nodes. Then the values of the three nodes on Forward Digit are pooled and weighted based on distance (the closest node has the highest weight) to calculate the participant's predicted score. The mean absolute difference between the predicted scores and true scores of the unseen sample was used as the measure of prediction error.

To better evaluate the average model performance, we repeated the cross‐validation process 1,000 times to derive distributions of the mean prediction errors. The distributions for chance level were achieved by randomly shuffling the order of the predicted scores, then subtracting the true scores to obtain a null mean absolute difference. For each iteration of the 1,000 cross‐validations, we also repeated the shuffling 100 times to create a null distribution containing 100,000 values of prediction errors. Finally, the mean prediction errors for all variables were compared to the corresponding null distributions to compute *p*‐values by calculating the proportion of the null distribution greater than the mean prediction error.

##### Assessing generalizability across samples

We were also interested in whether the representativeness of the SOM extended to other samples. To test this, we used a SOM trained on the entire CALM/ACE dataset to predict task scores in the pre‐ and post‐training datasets respectively. The CALM/ACE sample is much larger in size and included a wide range of ability levels. This means that a model based on these data is more likely to generalize well with other datasets. We generated the chance level distributions for pre‐ and post‐training samples similar to the last step by shuffling the order of predicted scores 100,000 times. Again, true prediction errors were compared to derive *p*‐values.

An alternative way to address this question is to compare prediction errors for the CALM/ACE sample and the pre‐ and post‐training samples respectively. If the SOM model represents the training study data as well as it does the CALM/ACE sample, then prediction errors should not differ from each other. For this purpose, we again repeated the same cross‐validation procedures 1,000 times but randomly removed 179 participants from the CALM/ACE sample each time to keep the number consistent with the size of the pre‐ and post‐training data. The remaining CALM/ACE data points were used to train a SOM and make predictions for the removed CALM/ACE participants, as well as the pre‐ and post‐training samples respectively. A permutation test followed to test for significance of the difference in prediction errors between CALM/ACE and the training data (i.e., CALM/ACE vs. pre; CALM/ACE vs. post). We also used permutation tests to compare the prediction errors of the pre‐training and post‐training data respective to one another.

#### 2.4.3 Does training alter the relationships between tasks?

Here we ask this question in two ways. Each model node is an instance of a multivariate task relationship that exists in the data used to train the SOM. If these SOM maps have less predictive power when used to estimate new data points this means that different multivariate task relationships exist in that dataset, which are not well accounted for by the model. This is the first way of testing whether the training has changed task relationships.

Secondly, we also addressed this question by comparing the SOMs trained on the pre‐ and post‐training datasets directly. If two tasks tap into similar cognitive processes, their model representations should overlap; if training alters task relationships, then the model representations of tasks in the SOM fit to pre‐training data should be substantively different to those in the SOM fit to post‐training data.

**Figure 2:**
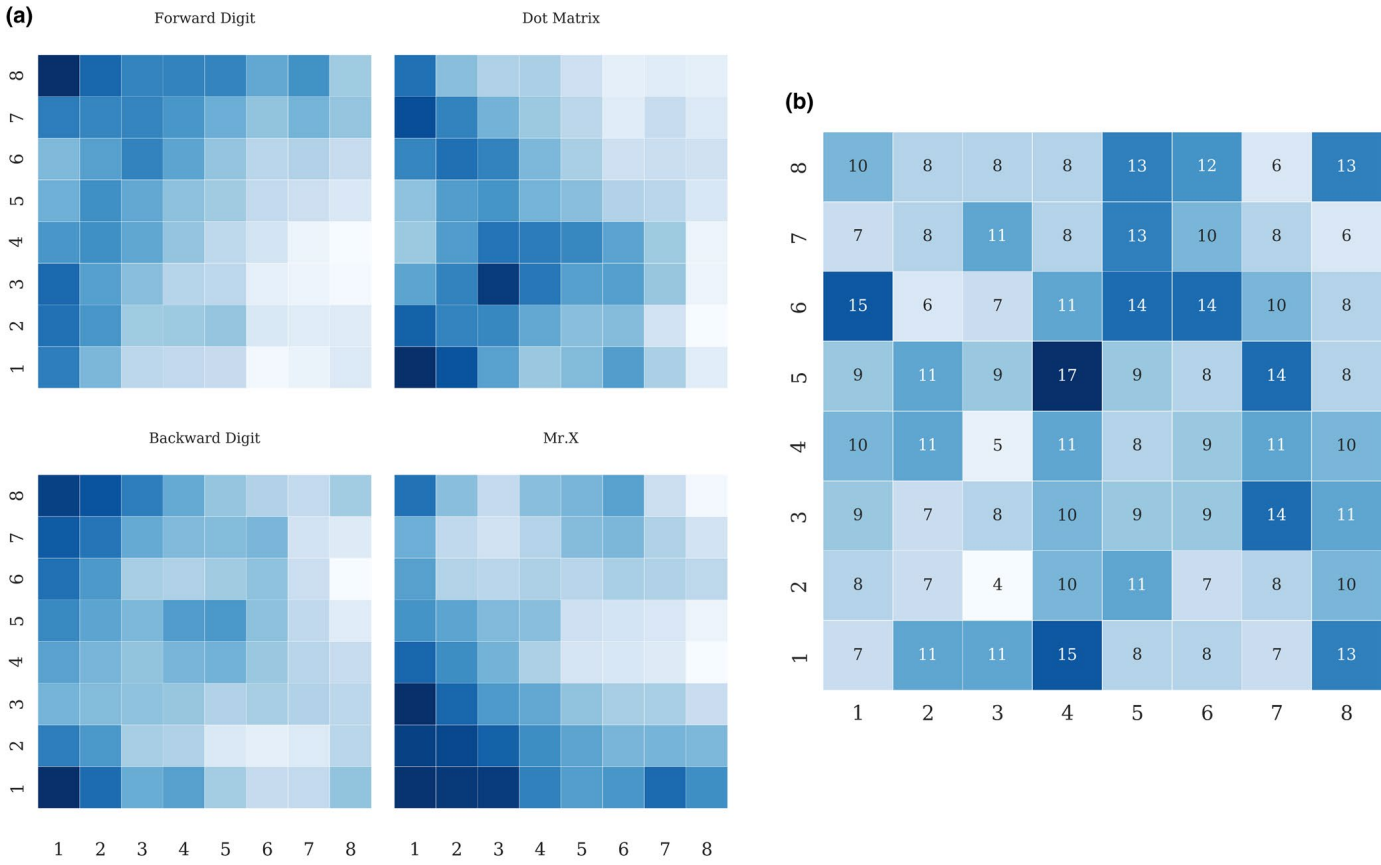
Overview of SOM model trained on CALM/ACE sample. (a) Visualization of node weights of the SOM (component planes) separated by each task. (b) Number of participants allocated to each node. Abbreviations: CALM/ACE, Centre for Attention, Learning and Memory/Attention and Cognition in Education; SOM, self‐organizing map

To access task similarities as represented by SOM, elements of SOM node‐weight vectors can be extracted individually (e.g., the first element of all node vectors) to form a “component plane.” Each plane corresponds to a representation of a task. The pairwise correlation coefficients between component planes can be derived and serve as multivariate activity patterns, which is useful for quantitative analysis. If two tasks tap into similar cognitive processes, their activity patterns ought to overlap (e.g., Figure 2 the Forward Digit and Backward Digit which both involve auditory information, share more topological similarity). By extracting the correlations between the same pair of tasks before and after training, we could then make a direct comparison of how their relationships had changed as a result of the training. To compute the relationship, component planes associated with each pair of tasks were compared using Pearson's correlation coefficient. Then, the similarity values are assembled into a 4 × 4 matrix.

Once the similarity matrices for pre‐ and post‐training were computed, we compared the same pairs of tasks between times of pre‐ and post‐training to identify any significant differences in correlation coefficients. We chose to bootstrap the node‐weight elements associated with the two tasks and computed the correlation coefficients before subtracting one from another (post‐training pairwise correlation—pre‐training pairwise correlation). By repeating this procedure 10,000 times, we obtained a distribution of the difference between correlations. If zero falls within the bottom or top 5% of the distribution, we reject the null hypothesis that the two correlation coefficients are not different, with a false‐positive rate of α = 0.05. We conducted this analysis for all pairs of tasks.

#### 2.4.4 Are there subgroups with different profiles of change following training?

*K*‐means clustering provides a data‐driven method for identifying *k* relatively homogenous subgroups within the SOM node‐weight vector space by minimizing the distance between data points and the centroids of each cluster (MacQueen, 1967). Although there is no clear theoretical rationale for the choice of number of clusters, in the Supporting Information we included multiple cluster solutions to demonstrate the resulting differences from various choices of *k*, and included a Silhouette Analysis of the different clustering solutions as a measure of the clustering quality.

We first identified subgroups within the SOM fit to the CALM/ACE data, by applying *K*‐means clustering to the node weights. Once nodes were grouped based on similarity, participants were allocated to the cluster to which their Best Matching Unit belongs to. This provided us with clusters of children based on nodes they were assigned to in the original mapping. This process was repeated 1,000 times, with the map retrained on every iteration; and the *K*‐means clustering recalculated to check that the clusters were robust. Participants in the training datasets were also allocated to these identified clusters in the same manner (i.e., based on closest Euclidean distance) at both pre‐ and post‐training, separately. Profiles of subgroups were characterized by calculating their respective means and standard errors on each of the tasks and compared between groups to identify the ways in which they differ. In the case of the cognitive training datasets, we also contrasted children who changed subgroup following the training. We did this by calculating gain scores (post‐ minus pre‐training) on each task as a way of testing how different gain scores are associated with changes in subgroup membership.

Finally, we tested whether these clusters were predicted by another measure that was not included in the SOM training or clustering, namely matrix reasoning scores. Importantly this is neither a baseline outcome assessment nor in the training regime. Scores on a matrix reasoning task taken from Wechsler's Abbreviated Scale of Intelligence (WASI; Wechsler, Scales, & Index, 2012) were available for 158 participants in the training sample. Matrix reasoning is considered a measure of general fluid intelligence (Gf), which refers to the ability to reason and solve novel problems. Gf is a critical factor for success in a wide variety of cognitive tasks and the capacity to learn in general (Gray & Thompson, 2004). We explored whether performance on the WASI matrix reasoning task assessed prior to training was predictive of change of subgroup membership.

#### 2.4.5 Summary

The above pipeline describes our stepwise analyses. (a) SOMs were used to model task relationships. We cross‐validated the model trained on the large CALM/ACE sample and tested its representativeness with the pre‐ and post‐training samples. (b) SOM models representing pre‐ and post‐training samples were compared directly by training new SOMs with the two samples and then comparing them through a representational dissimilarity analysis that examined how task relationships changed following training. (c) *K*‐means clustering was used to identify relatively homogeneous cognitive profiles in the CALM/ACE sample as represented by the SOM model. (d) Participants in the training dataset were subsequently mapped to these subgroups to investigate the changes in these profiles as a function of training. (e) We tested whether fluid intelligence predicted the change in profiles following training.

## 3 RESULTS

A 64‐node (8 × 8) SOM with an initial neighbourhood size of 2 was trained over 10 ordering phase steps and two fine‐tuning phase steps using the CALM/ACE data (Quantization error = 9.72); quantization error is defined as the mean absolute distance between the input vectors (i.e., training data) and their corresponding Best Matching Units. The rationale behind the selection of these parameters, alongside different solutions with different parameters, is included in the Supporting Information. Figure 2 shows how the SOM represents the four tasks as well as the number of participants allocated to each node.

### 3.1 Does the SOM model represent the samples well?

We first cross‐validated the model performance of SOM trained on the CALM/ACE data using permutation testing. The SOMs proved capable of predicting unseen CALM/ACE data significantly better than chance for all four task variables (Table 2).

**Table 2:** SOM prediction errors for CALM/ACE sample, pre‐ and post‐training sample respectively

		Forward Digit	Dot Matrix	Backward Digit	Mr. X
CALM/ACE	Prediction error (standard score)	11.68	11.57	9.41	11.91
	*p*	<0.001***	<0.001***	<0.001***	<0.01**
Pre‐training	Prediction error (standard score)	13.41	12.24	11.83	14.13
	*p*	<0.001***	<0.001***	<0.001***	<0.001***
Post‐training	Prediction error (standard score)	14.25	17.67	12.12	14.71
	*p*	<0.001***	<0.001***	<0.001***	<0.001***

Note

Prediction error was defined as mean absolute difference between the predicted scores and true scores. *p‐*values were derived from comparing the prediction errors against the corresponding chance level distributions. The chance levels were achieved by randomly shuffling the order of the predicted scores, then subtracting the true scores for 100 times within each cross‐validation literation, to obtain a null distributions of mean absolute difference.

Abbreviations: ACE, Attention and Cognition in Education; CALM, Centre for Attention, Learning and Memory; SOM, self‐organizing map.

** *p* < 0.01.

*** *p *< 0.001.

Next, a SOM trained on the entire CALM/ACE sample was then used to test how well it represents the pre‐ and post‐training datasets, again using the same method. The model predicted unseen data from other samples better than chance on all tasks.

We also directly compared the CALM/ACE prediction errors with the pre‐ and post‐training data (Table 3). Predicting the remaining CALM/ACE sample was more accurate than predicting the pre‐ or post‐training samples. A direct comparison of the pre‐ and post‐training prediction accuracies revealed comparable prediction accuracies on all tasks except on the Dot Matrix task, wherein the prediction accuracy dropped significantly for the post‐training sample.

**Table 3:** Direct comparisons between SOM prediction errors between unseen CALM/ACE and pre‐training sample (CALM/ACE vs. pre‐training), as well as between CALM/ACE and post‐training (CALM/ACE vs. post‐training), and between the pre‐training and post‐training (pre vs. post‐training)

		Forward Digit	Dot Matrix	Backward Digit	Mr. X
CALM/ACE versus pre‐training	Prediction error difference	1.73	0.66	2.42	2.21
	*p*	<0.001***	0.177	<0.001***	<0.001***
CALM/ACE versus post‐training	Prediction error difference	2.57	6.09	2.70	2.80
	*p*	<0.001***	<0.001***	<0.001***	<0.001***
Pre‐ versus post‐training	Prediction error difference	0.83	5.43	0.28	0.59
	*p*	0.201	<0.001***	0.373	0.296

Note

*p*‐values were derived from permutation tests.

Abbreviations: ACE, Attention and Cognition in Education; AWMA, automated working memory assessment; CALM, Centre for Attention, Learning and Memory.

*** *p* < 0.001.

### 3.2 Does training alter the relationships between tasks?

**Figure 3:**
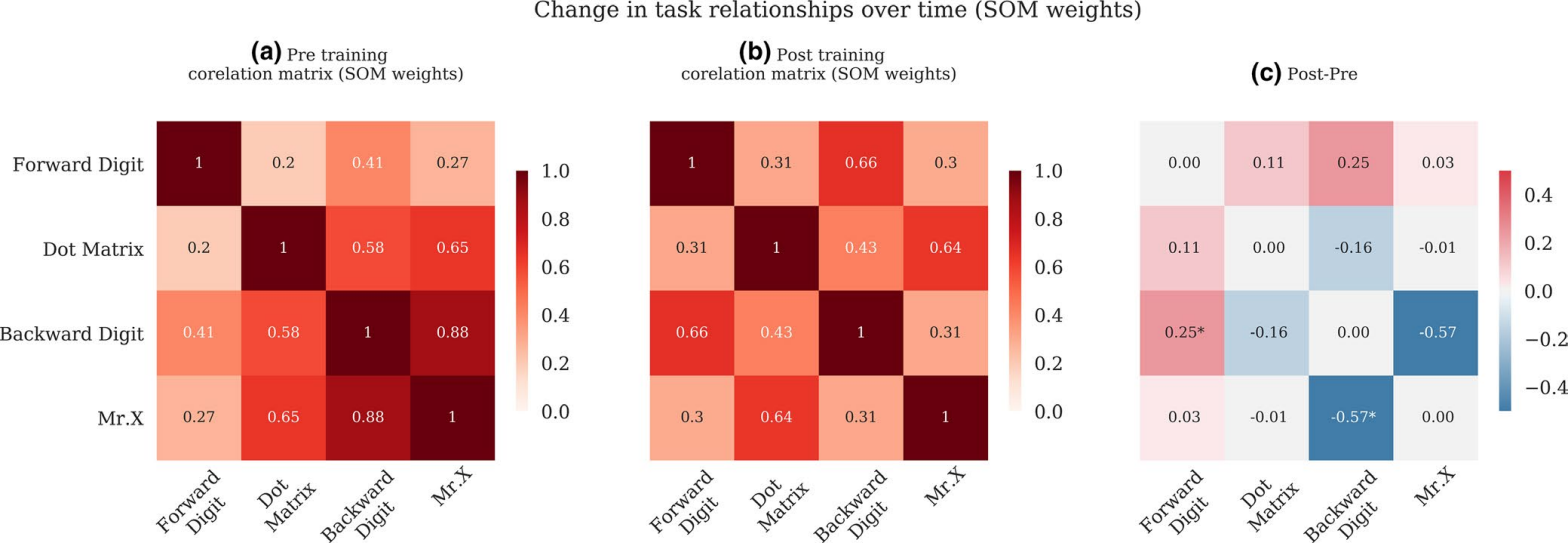
Pairwise task relationships derived from SOM weights before and after training and the difference over time. Larger value indicates more similarity between the two tasks. (a) Task relationships for pre‐training sample. (b) Task relationships for post‐training sample. (c) Difference in similarity between pre‐ and post‐training (post–pre). The Backward Digit‐ Mr. X pair showed significant change after training (*p* < 0.01), as did the Forward Digit‐Backward Digit pair (*p *< 0.05). Abbreviation: SOM, self‐organizing map

New SOMs trained on pre‐ and post‐training data respectively were compared to examine changes in task relationships as a function of training. Pairwise correlation coefficients were computed from the SOMs component planes representing tasks and assembled into similarity matrices. Figure 3a,b depict the pre‐ and post‐training matrices. We conducted pairwise comparisons before and after the training to understand whether there are specific alterations between any of the task relationships. Permutation testing indicated a significant difference in the Backward Digit‐MR.X pair (*p *< 0.01) and in the Forward Digit‐Backward Digit pair (*p *< 0.05). In other words, the way tasks are represented in the SOM weights changes following training, with some becoming more similar and others more dissimilar (we refer the readers to Figure S10 and the section titled “Comparison with the control group” in the Supporting Information for the same analysis in the control data).

### 3.3 Are there subgroups with different profiles of change following training?

**Figure 4:**
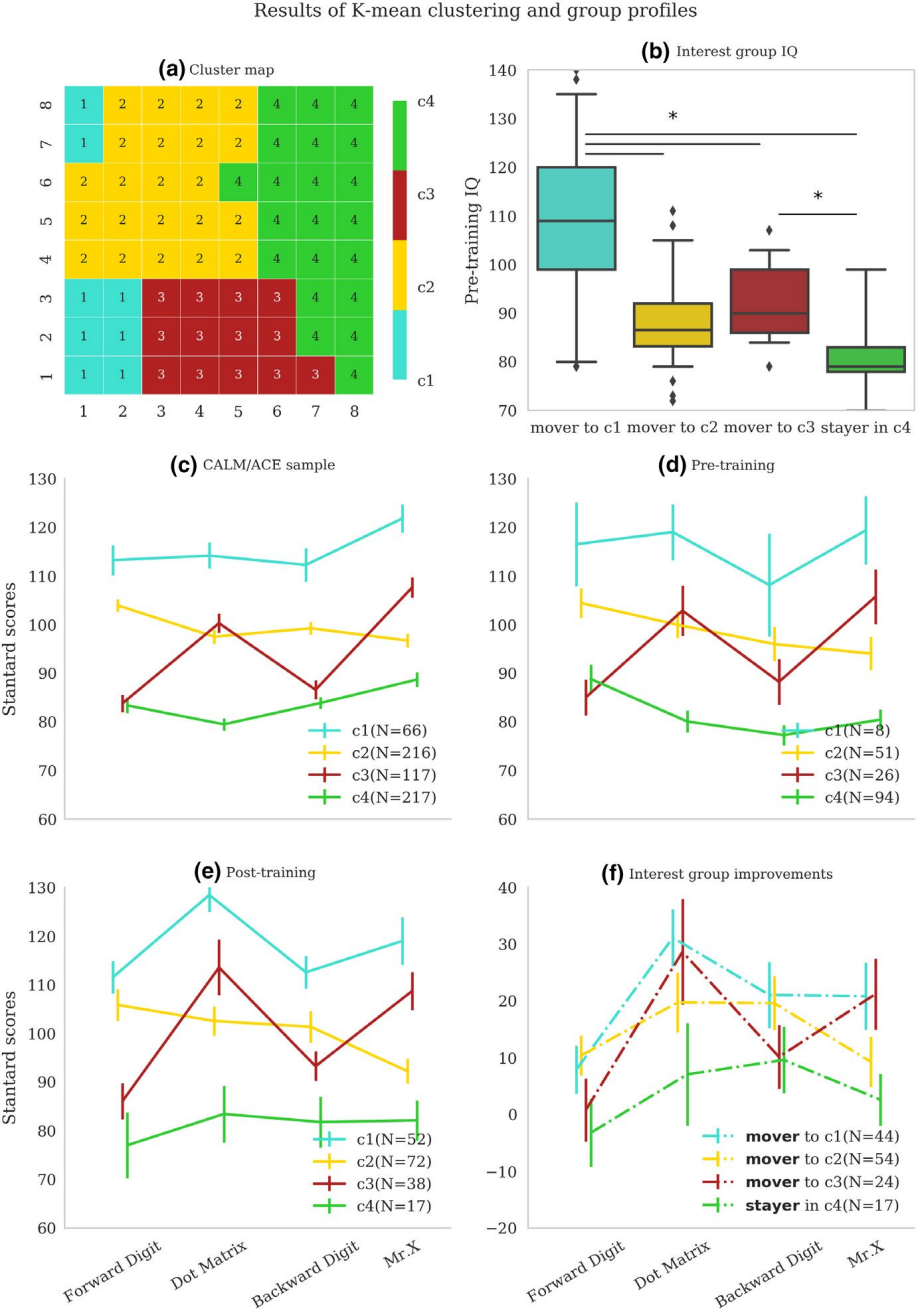
Results of *K*‐means clustering and comparison of subgroup profiles. (a) SOM nodes were partitioned into four clusters. (c–e) Comparison of task scores among the subgroups in the CALM/ACE, the pre‐training and the post‐training sample respectively. Error bar indicates 95% confidence interval. (f) Comparison of improvement profiles of the interest groups: participant who moved to the highest‐performing group (Cluster 1) after training, those who moved to the medium group with verbal‐specific gains (Cluster 2), those who moved to the medium group with visuospatial‐specific gains (Cluster 3), and those who stayed in the low‐performing group (Cluster 4). For clarity, the first three interest groups only included participants who moved to the respective group from the outside, not those that were already there at pre‐training, and vice versa for the fourth interest group. (b) WASI matrix reasoning score of the three interest groups. *p < 0.05. Abbreviations: CALM/ACE, Centre for Attention, Learning and Memory/Attention and Cognition in Education; SOM, self‐organizing map; WASI, Wechsler's Abbreviated Scale of Intelligence

We applied *K*‐means clustering to the node‐weight vector space pertaining to the SOM trained on the CALM/ACE data using a *K* = 4 (see the Supporting Information for robustness of clustering quality across different *K*s). The resulting partition of the SOMs nodes can be seen in Figure 4a. Participants were allocated to the cluster to which their Best Matching Units belonged. Profiles of subgroups were characterized by calculating the respective means and standard errors on all four tasks in the CALM/ACE sample (Figure 4c).

Between‐group ANOVAs were conducted for each task and all indicated significant differences (Forward Digit: *F*(3, 612) = 233.17; Dot Matrix: *F*(3, 612) = 244.03; Backward Digit: *F*(3,612) = 175.59; Mr. X: *F*(3, 612) = 179.41; all *p* < 0.001). Results from the post‐hoc Tukey's HSD tests showed that all subgroups differed significantly from one another at the 0.05 level except for Groups 2 and 3 on Dot Matrix and Groups 3 and 4 on Forward Digit and Backward Digit. The algorithm identified a subgroup of participants who achieved a high level of performance on all tasks, a subgroup whose scores were at the lower end of the distribution and two subgroups who were in the middle. One of these middle subgroups tended to have average performance on all tasks, whereas the other tended to have average or slightly above average performance on the visual spatial tasks but below average performance on the verbal tasks.

Participants in the pre‐ and post‐training sample were also allocated to one of the four identified subgroups (Figure 4d,e). The profiles of the training study participants in each subgroup were similar to those of the CALM/ACE sample, highlighting the ability of *K*‐means clustering to determine relatively homogenous groups (see Supporting Information). Specifically, ANOVAs indicated significant group differences across all measure for pre‐ and post‐training (pre‐training: Forward Digit: *F*(3, 175) = 28.58; Dot Matrix: *F*(3, 175) = 69.84; Backward Digit: *F*(3, 175) = 40.76; Mr. X: *F*(3, 175) = 55.67; all *p* < 0.001; post‐training: Forward Digit: *F*(3, 175) = 50.31; Dot Matrix: *F*(3, 175) = 58.08; Backward Digit: *F*(3, 175) = 33.63; Mr. X: *F*(3,175) = 55.18; all *p* < 0.001). Post‐hoc Tukey's HSD tests revealed all pairwise groups were significantly different at the 0.05 level except for Groups 2 and 3 on Dot Matrix and Groups 3 and 4 on Forward Digit in the Pre‐training dataset; difference between Groups 1 and 2 on Forward Digit was marginally non‐significant (*p* = 0.06). For the post‐training, all subgroups were different from one another.

We also contrasted children who moved to a different subgroup following training and calculated their gain scores (post‐training minus pre‐training scores) to capture individual differences in training‐related improvement. Four interest groups were identified (separate from but related to the original four clusters), namely: children who moved to the highest performance group (mover to Cluster 1), children who moved to Group 2, children who moved to Group 3 and children who stayed in the lowest performance group (stayer in Cluster 4). For clarity, the first three interest groups only included participants who moved to the respective group from the outside, not those that were already there at pre‐training, and vice versa for the fourth interest group. The gain scores of these groups are shown in Figure 4f. These groups were significantly different from each other according to ANOVA (Forward Digit: *F*(3, 122) = 4.94; Dot Matrix: *F*(3, 122) = 7.14; Backward Digit: *F*(3, 122) = 6.34; Mr. X: *F*(3, 122) = 8.57; all *p* < 0.001) and post‐hoc tests (see Table 4 for multiple pairwise comparison results). Overall, movers to 1 had the highest improvement across all measures compared to the other groups. Movers to 2 were characterized by moderate gains globally but benefited less on Dot Matrix and Mr.X relative to children moved to Cluster 1. The third group, children moved to Cluster 3 had comparable magnitude of gains on Dot Matrix and Mr.X to movers to 1, but significantly less gains on Forward and Backward Digit tasks than movers to 1 or 3. Unsurprisingly, children who stayed in the lowest performance group (Cluster 4) had overall limited benefit from the training.

**Table 4:** Results of multiple comparison between different improvement profiles

	Movers to C1	Movers to C2	Movers to C3	Stayers in C4
Forward Digit				
Movers to C1	NA	*p* = 0.88	*p* = 0.36	*p* < 0.05*
Movers to C2		NA	*p* = 0.09	*p* < 0.01**
Movers to C3			NA	*p* = 0.63
Stayers in C4				NA
Dot Matrix				
Movers to C1	NA	*p* = 0.08	*p* = 0.90	*p *< 0.001***
Movers to C2		NA	*p* = 0.18	*p* = 0.06
Movers to C3			NA	*p < *0.01**
Stayers in C4				NA
Backward Digit				
Movers to C1	NA	*p* = 0.71	*p* < 0.01**	*p* < 0.01**
Movers to C2		NA	*p* < 0.05*	*p* = 0.06
Movers to C3			NA	*p* = 0.9
Stayers in C4				NA
Mr. X				
Movers to C1	NA	*p* < 0.01**	*p* = 90	*p* < 0.001***
Movers to C2		NA	*p* = 0.05	*p* = 0.30
Movers to C3			NA	*p* < 0.01**
Stayers in C4				NA

* *p* < 0.05.

** *p* < 0.01.

*** *p* < 0.001.

To investigate whether performance on a measure of fluid intelligence (WASI matric reasoning) could predict individual differences in patterns of improvement, we compared the four interest groups’ WASI scores assessed prior to training (see Figure 4b). ANOVA indicated significant WASI score difference among the groups (*F*(3, 122) = 28.83, *p* < 0.001). Post‐hoc tests showed that movers to Cluster 1 had higher WASI scores (*M* = 108.72, *SD* = 17.39) than movers to Cluster 2 (*M* = 88.46, *SD* = 8.70, *p *< 0.001), movers to Cluster 3 (*M* = 92.31, *SD* = 7.94, *p *< 0.001), and stayers in Cluster 4 (*M* = 81.76, *SD* = 8.72, *p *< 0.001). Movers to 3 also had higher WASI scores compared to stayers in 4 (*p* < 0.05).

## 4 DISCUSSION

Our understanding of cognitive training has hitherto focused on exploring its impact on single tasks (though with some notable exceptions, e.g., Guye et al., 2017; Karbach et al., 2017; Schmiedek et al., 2010) and treating all participants as a single homogenous group (e.g., Borella et al., 2014; Bürki et al., 2014; Guye et al., 2017; Zinke et al., 2014). In the present study, we used machine learning to show that WM training alters the relationships between tasks, implying that the cognitive processes recruited for performing those tasks can change following training. Furthermore, we identified subgroups with differential responses to training which were predicted by fluid intelligence scores.

### 4.1 SOMs accurately represent task relationships

A SOM was fit to a large dataset of children who were assessed on standardized measures of verbal and visuospatial short‐term and WM. Using leave‐N‐out cross‐validation, we showed that SOMs fitted on these data predicted performance on unseen data for all tasks. These predictions generalized to the cognitive training samples; importantly, however, the model fit and prediction accuracy was reduced significantly following training for the Dot Matrix, the implication of which is discussed subsequently.

### 4.2 Task relationships change following training

Multiple studies have shown that performance on individual tasks improves following training (for reviews, see Hertzog et al., 2008; Melby‐Lervag et al., 2016; von Bastian & Oberauer, 2014). But this does not provide any insight into whether or how underlying constructs are being changed, or whether different cognitive processes are recruited following the intervention. One way of investigating this is to test whether relationships between tasks change as a function of training. Following training, we identified a large decrease in prediction accuracy for the Dot Matrix task mirroring substantial improvements in task performance. Lower prediction accuracy following training also suggests that the relationships between Dot Matrix and the other tasks may have been altered. In other words, new task relationships (i.e., multivariate data points) exist in the post‐training data that were not learnt or represented in a large sample of children who did not complete cognitive training. In this case, the training program contains many exercises similar to the Dot Matrix task (i.e., visuospatial serial recall, Klingberg et al., 2005), and thus subjects may show a more task‐specific effect rather than a domain‐general improvement. This is in line with demonstrations of maximal transfer to assessment tasks most similar to those trained (Gathercole et al., 2019), with highest levels of transfer for tasks with the greatest numbers of shared task features (Soveri, Antfolk, Karlsson, Salo, & Laine, 2017). If the bulk of improvements had been domain general, then we would expect similar‐sized improvements on other tasks measuring visuospatial WM (i.e., Mr X), but these improvements were relatively small. Moreover, this is further exaggerated when we look at the size of improvements relative to those in the control group, indicative of practice effects. These changing task relationships underscore the fact that the cognitive processes we recruit for individual tasks are not static but can change as a function of experience.

Most task relationships remained stable across training, but the correlations between MR.X‐Backward Digit and Forward Digit‐Backward Digit changed significantly. The MR.X‐Backward Digit pair substantially decreased in correlation following training, whereas there as a moderate increase for the Forward Digit‐Backward Digit pair. Again, this shows that relationships between tasks, as represented by the SOM, are subject to change following training. One possibility is that as subjects practice the Backwards Digit task—a version of which exists in the training battery—they gradually start to recruit similar cognitive processes or strategies that they previously used for the Forward Digit task, like chunking. The end result is that the SOM represents these tasks more similarly following training. By contrast, the task is now represented more differently to the other complex span task in the assessment battery, Mr X. In short, even though both Backwards Digit and Mr X are described as WM tasks, and both improve overall following training, the changing way that they are represented by the SOM indicates that different cognitive processes or strategies are recruited for them following training. Importantly this would not be captured by a conventional approach to testing for transfer.

### 4.3 Subgroups with different training profiles

There is an increasing interest in individual differences in cognitive training effects. The approach typically taken is to explore the impact of known factors, like age (Borella et al., 2014; Schmiedek et al., 2010), baseline ability (Bürki et al., 2014; Guye et al., 2017; Zinke et al., 2014) or cognition‐related beliefs (e.g., malleability of intelligence; Jaeggi et al., 2014), on training‐related gains. Here we explored individual differences in a different way, by identifying subgroups in the training profiles themselves. Clustering identified four groups that differed in their performance across tasks (High, Medium [visuospatial and verbal profiles], and Low). Changes in group membership after training were associated with the magnitude, and patterns of, gain scores. This suggests there are differential improvement trajectories among children, which would be lost in conventional group‐level comparisons. These improvement profiles were meaningfully associated with fluid intelligence: those who made the largest improvements across all measures (movers to the highest performing group) had significantly higher fluid reasoning skills compared with those who stayed in one of the low‐performing groups. General intelligence is thought as the ability to reason and solve novel problems (Duncan & Owen, 2000), or an index for flexible cognitive resources believed to play a critical role in the process of decomposing the unfamiliar tasks into their component parts (Duncan, Chylinski, Mitchell, & Bhandari, 2017). This may indicate that the ability to abstract and generalize newly learned routines to unpractised tasks is one of the deciding factors of transfer effects (Gathercole et al., 2019).

The positive association between fluid intelligence and improvement profile is reminiscent of some previous studies that have shown age‐related and ability‐related magnification effects in the context of cognitive training (e.g., Bürki et al., 2014; Guye et al., 2017). Magnification effects are more typically observed in the context of strategy‐based training than process‐based training (e.g., Karbach & Verhaeghen, 2014; Karbach et al., 2017), possibly indicating that the training intervention in this study facilitated strategy acquisition (Guye & von Bastian, 2017). Indeed, it has been shown that training‐related improvements in WM may be mediated by implicit development of task‐specific strategies such as grouping of sequential information for recall (Dunning & Holmes, 2014; Minear et al., 2016). Gathercole et al. (2019) argue that these kinds of effects are evidence that training‐related gains rely on the construction and refinement of new cognitive routines and strategies. Individuals with higher levels of cognitive performance at baseline may have more capacity to acquire and perform strategies that enhance the training effect (Lövdén et al., 2012). Our findings would support this. An interesting line of enquiry would be to investigate whether children with relatively low intelligence scores could benefit from explicit instructions to help aid strategy generation while training.

### 4.4 General discussion

We show that task relationships change following training (according to two separate measures), thereby indicating that the underlying mechanisms tapped by training might be task‐specific rather than domain‐general, and subject to change over time. We have also demonstrated that task performance trajectories are subject to individual differences under this paradigm. This highlights the need to reconsider the interpretation of training‐related gains. Children could improve significantly on a particular task via learning specific strategies whilst having moderate or no gains on other tasks claimed to measure the same construct (Moreau, Kirk, & Waldie, 2016).

To remedy this, previous studies investigating the training‐induced improvement on the ability level used latent factor analysis, which is necessarily constrained by how the observed variables load onto the latent factors before and after the training for the sake of model comparability and interpretability (Bürki et al., 2014; Guye et al., 2017; Karbach et al., 2017; Lövdén et al., 2012; Schmiedek et al., 2010). However, this assumption is challenged by the current findings, which imply that training does not only enhance the performance, but also alters the task structures. In the Supporting Information, we show that this is indeed the case in the context of the current dataset, by fitting linear models to the data. The difference in best fitting model before and after training could either be due to the enhancement of task‐specific processes or an increase in individual variance across tasks, or both. Either way, it suggests that the best latent variable model before and after the training may not necessarily be the same. Fitting different models pre‐ and post‐training would limit the meaningfulness of comparisons across time points (Dimitrov, 2006). Conversely, imposing parameter invariance when the real data suggest otherwise could lead to a large estimation bias of the model, which cannot be reliably indicated by fit statistics (Clark, Nuttall, & Bowles, 2018). If such cases arise, the SOM approach taken here is a potentially more flexible alternative that does not rely on as many assumptions, while still allowing for meaningful comparisons over time.

Importantly, our findings may be specific to the set of training and assessment tasks we had available. Moreover, our dataset was a composite of many individual studies, with independent recruitment criteria. Nonetheless, our primary aim was to demonstrate a proof of principle, with potential benefits for those exploring multivariate profiles of change. The next step is for this to be tested in well‐powered training studies with a broader set of assessments.

## 5 CONCLUSION

SOM models provide an effective alternative for the representation and prediction of multivariate data typically found in training studies. Applying SOMs to the current training data revealed nuanced task relationships that are subject to change following WM training, suggesting that the underlying cognitive mechanisms of improvement may be at least partially task‐specific rather than domain‐general. The use of *K*‐means clustering revealed distinct subgroups with differentiable improvement trajectories. These improvement trajectories were related to pre‐training fluid intelligence.

## CONFLICT OF INTEREST

The researchers declare that there is no conflict of interest involved in this work.

## Data Availability

The data that support the findings of this study are available from the corresponding author upon reasonable request (Dr. Duncan E. Astle: duncan.astle@mrc-cbu.cam.ac.uk).
