# Supplementary material for: Mapping differential responses to cognitive training using machine learning
Source: Dev Sci. 2019 Jul 22;23(4):e12868. doi: 10.1111/desc.12868 (PMC7314597; doi:10.1111/desc.12868)
Supplement: Supplementary file 1 [file DESC-23-e12868-s001.docx]

# Supplementary material

## Selection of SOM parameters

We tested a range of SOM parameters provided by the Matlab 2017a Neural Network Toolbox. These included the map size, initial neighbourhood size, ordering phase steps and fine-tuning phase steps. We used a composite of quantization error and prediction error to evaluate each combination of the parameters within the tested range. Quantization error is defined as the mean absolute distance between the input vectors (i.e. training data) and their corresponding Best Matching Unit (BMU), which is an indicator of how well the model represents the input data. As discussed in the Method section, prediction error, defined as the mean absolute distance between the predicted and true values from the reserved testing data, is an indicator of the model’s ability to generalise to unseen data. Hence, we combined these two measures towards the aim of representing the input data whilst maintaining generalisability. Specifically, quantization errors and the mean prediction errors across the 4 cognitive measures were standardized with respect to their own distribution, achieved from all possible combinations of model parameters within the testing range, before being summed. For each combination of parameters, the results averaged over 100 iterations were used. The range of each parameter tested over are as follows:

- Map size: 6, 8, 10
- Initial neighbourhood size: 2, 3, 4
- Ordering (rough training) phase steps: 2, 4, 6, 8, 10, 20, 30
- Fine-tuning phase steps: 1, 2, 3

Fig S1 captures some of the consequences of different parameter combination selections with regards to prediction error, quantization error, and composite score respectively. The effect of SOM map size (i.e. number of nodes) exhibited the most influence on the former two measures (Fig S1a and S1b) which were in opposite directions: larger map led to lower quantisation but higher prediction error. On the other hand, the effect of ordering phase was only distinguishable below the range of 10 iterations. As such, it was not surprising that the composite score demonstrates a more ambiguous picture. We chose the combination of map size =10, ordering phase =10, fine-tuning phase = 2, initial neighbourhood = 2 as it produced the least composite error in the range of parameters we initially tested. Subsequently, we extended the range tested over to include ordering phase steps of 2,4,6,8 and 15. This revealed 2 parameter combinations that were marginally better but most likely negligible with regards to how they affect the findings in the paper.

**Figure S1. Overview of (a) mean prediction error and (b) quantisation error and (c) composite score as function of SOM training parameters (note that lower scores represent better outcomes).**

**(a)**


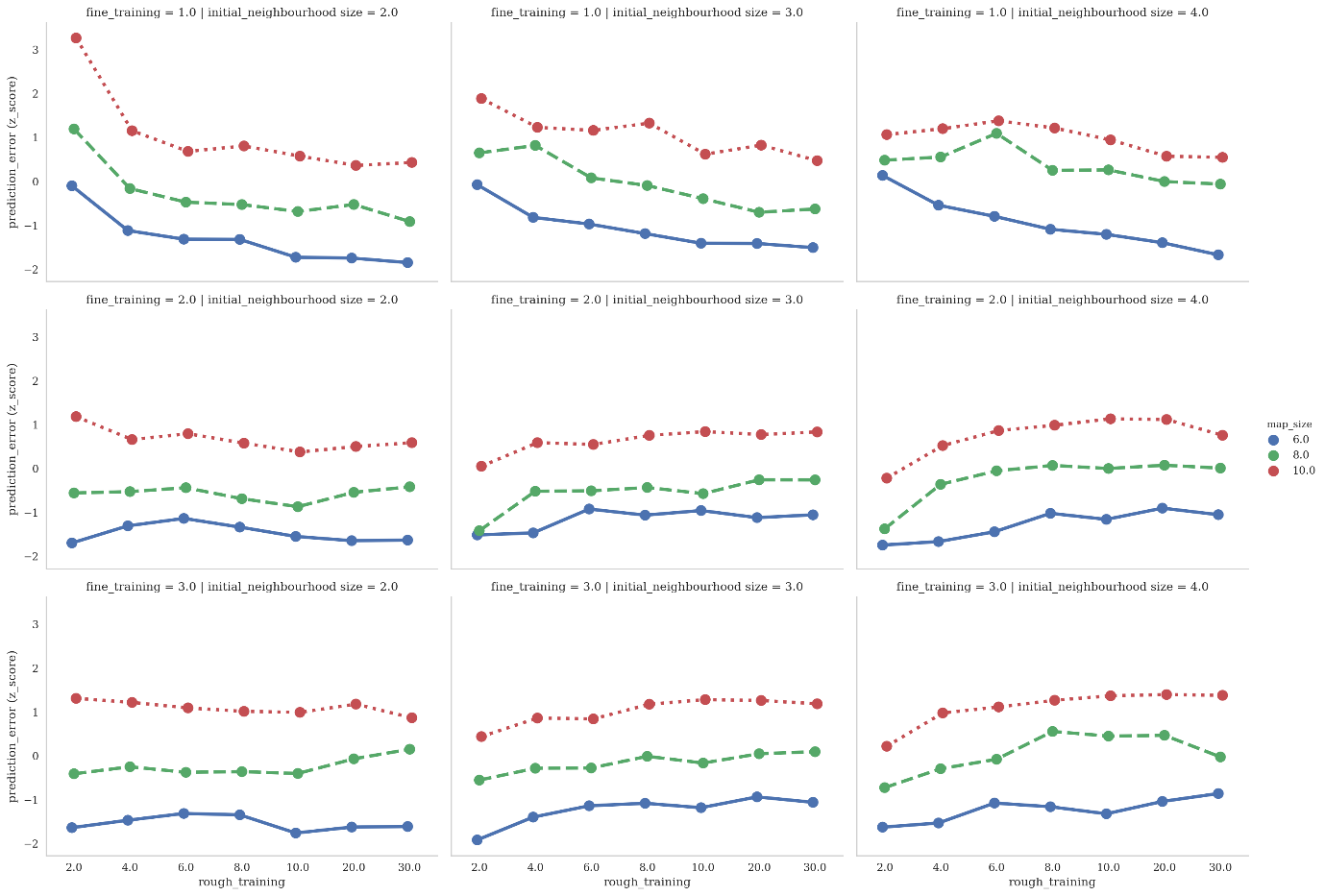


**(b)**


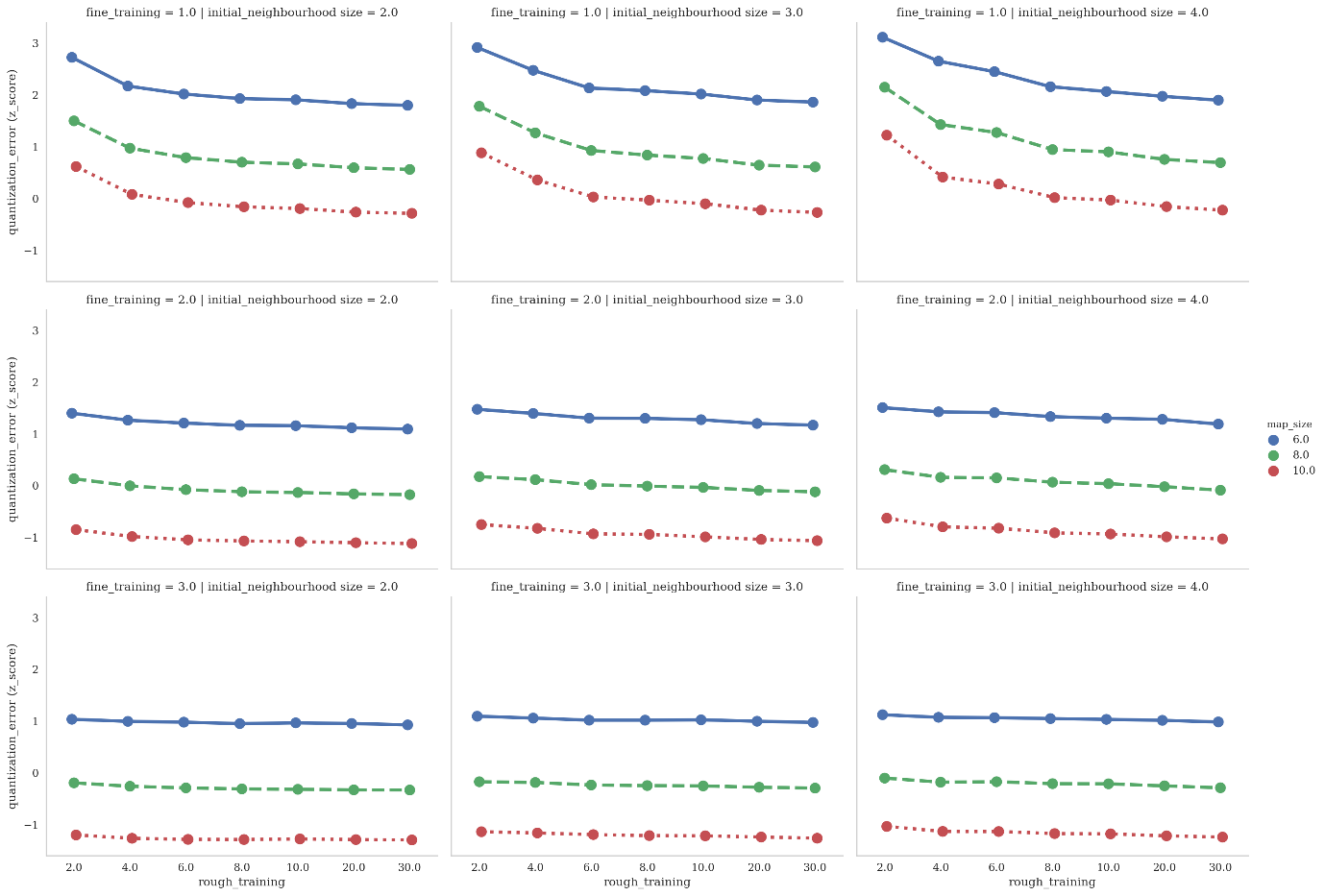


**(c)**


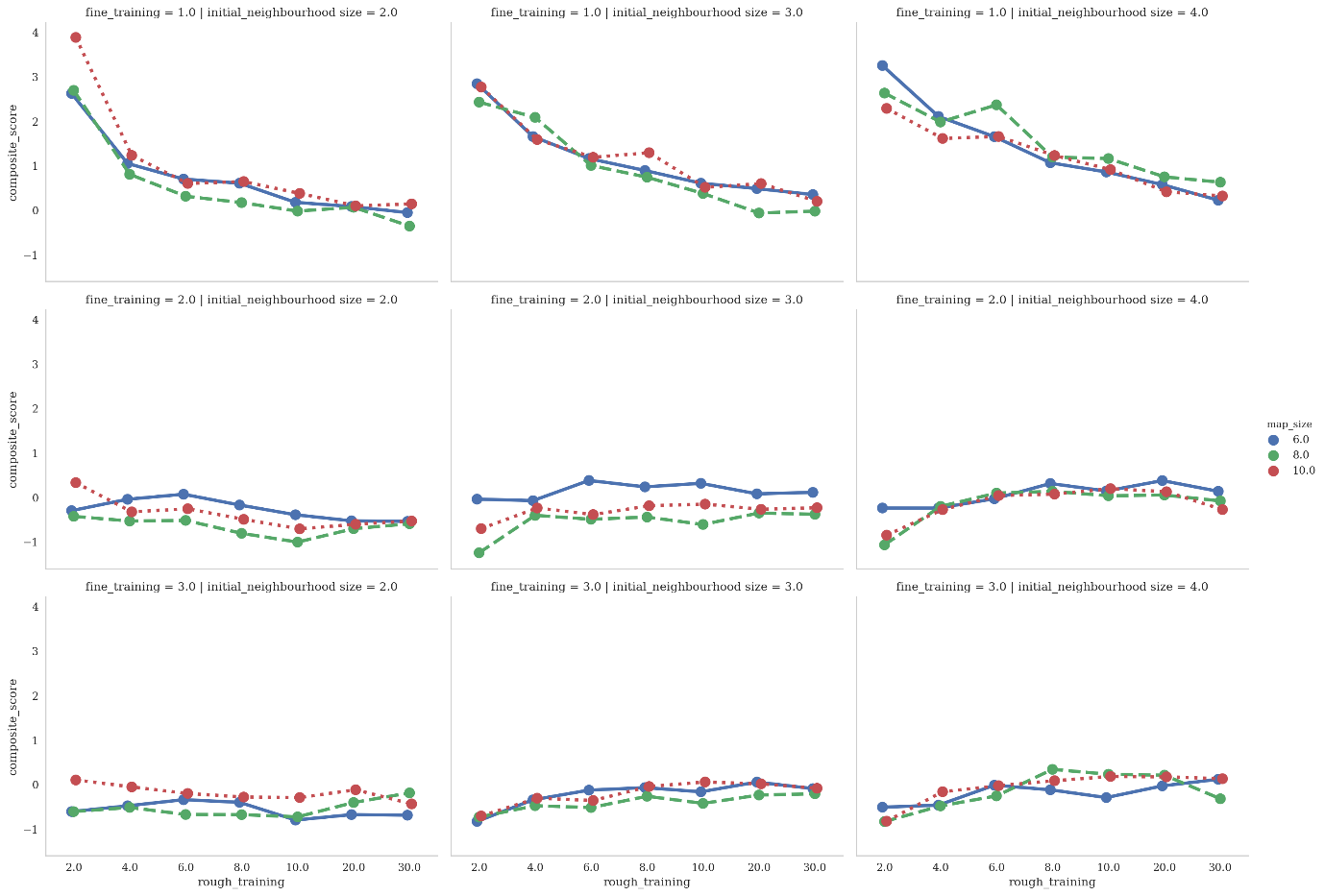


## Selection of K in identifying subgroups with differential profiles

The choice of K is somewhat arbitrary but could potentially influence the cognitive profiles associated with each subgroup. We chose K=4 as it resulted in statistically meaningful grouping of participants, as well as relatively homogenous improvement trajectories in the cognitive training data. Below are several figures that show the resulting profiles for different numbers of K and their resulting silhouette values (a way of determining robustness of grouping). We also show the resulting profiles from using a K of 4 upon the Pre and Post training data, the results of which show that despite some variation they are somewhat similar. Further, we include a comparison between the K-means on the SOM-weights vs those on the raw data. The silhouette coefficient is a measure of how close each data point is to its own cluster compared to the neighbouring clusters and thus provides a way to assess parameters like the number of clusters visually. This measure has a range of [-1, 1].

Silhouette coefficients near +1 indicate that a point is far away from the neighbouring clusters and therefor properly clustered. A negative value indicates that those samples are not robustly clustered. As Figure S2 shows, applying k-means clustering on the raw dataset results in a lower average silhouette coefficient compared to SOM weights, and some individuals were assigned to a ‘bad’ subgroup. This highlights the fact that the SOM reduces the noise in the data, thereby making the clustering more robust.

As can be seen in the Figure S3, a two cluster solution was favourable in terms of robustness. However, we opted for a four cluster solution in the end as it also appeared a reasonably stable solution, whilst also allowing us to capture more information and nuance with regards to the task performance profiles in the data (see figure S4).

Although we didn’t do an in depth analysis, you can see that the profiles derived from a clustering solution applied *directly* to the SOM models trained on the Pre-Training data (Figure S5a) are quite distinct from those derived from the CALM/ACE data (see main text Figure 4d). This suggests that the two samples are somewhat different in nature, but this difference may in part be an artefact of biased sample size, since the ACE dataset consisted of 90 typically developing children whose cognitive profiles were likely to be absent in the pre-training population as a result of the inclusion criteria. In fact, it can be seen that the scores of the highest-performing group in Figure S5a were on average lower, but the group size bigger than those of the highest-performing group in Figure 4d. In other words, pre-training sample lacks the high performance profiles that existed in the CALM/ACE and post-training samples. On the contrary, profiles derived from a clustering solution applied to the SOM map trained on the Post-Training data (Figure S5b), are similar versions of those derived from the CALM-ACE data clusters (see Figure 4e).

**Figure S2. Silhouette values for each cluster (K = 4) and the averaged silhouette coefficient (orange line) on SOM weights and raw CALM/ACE data respectively.**


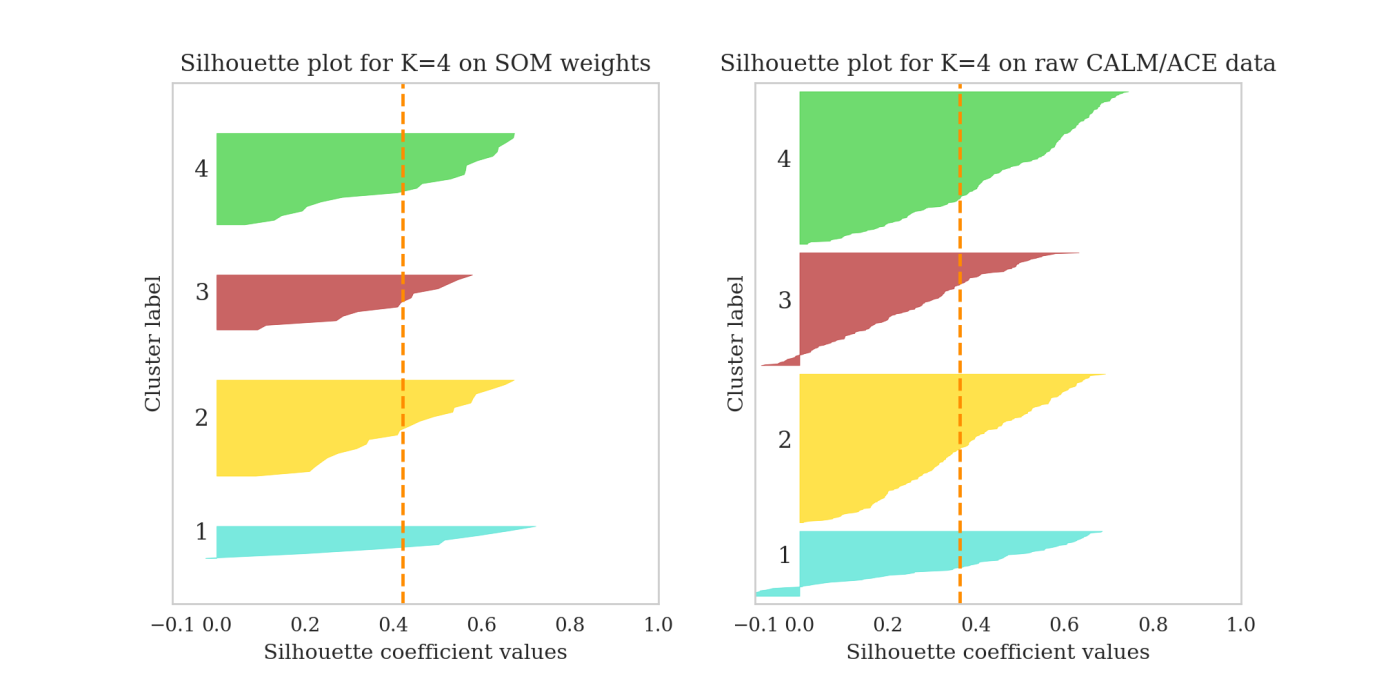


**Figure S3. Mean Silhouette coefficients for Choices of K ranging from 2-8:**

**
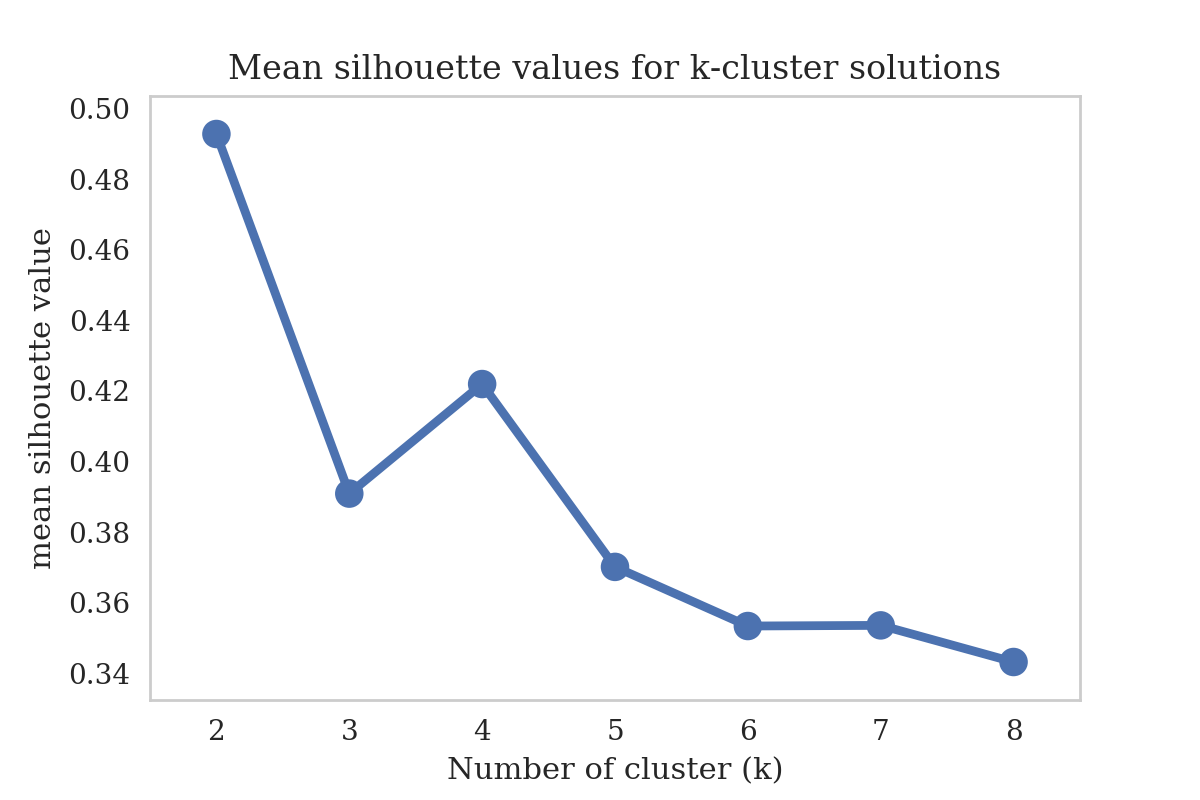
**

**Figure S4. Task performance profiles in the CALM-ACE dataset for differing number of K:**

**
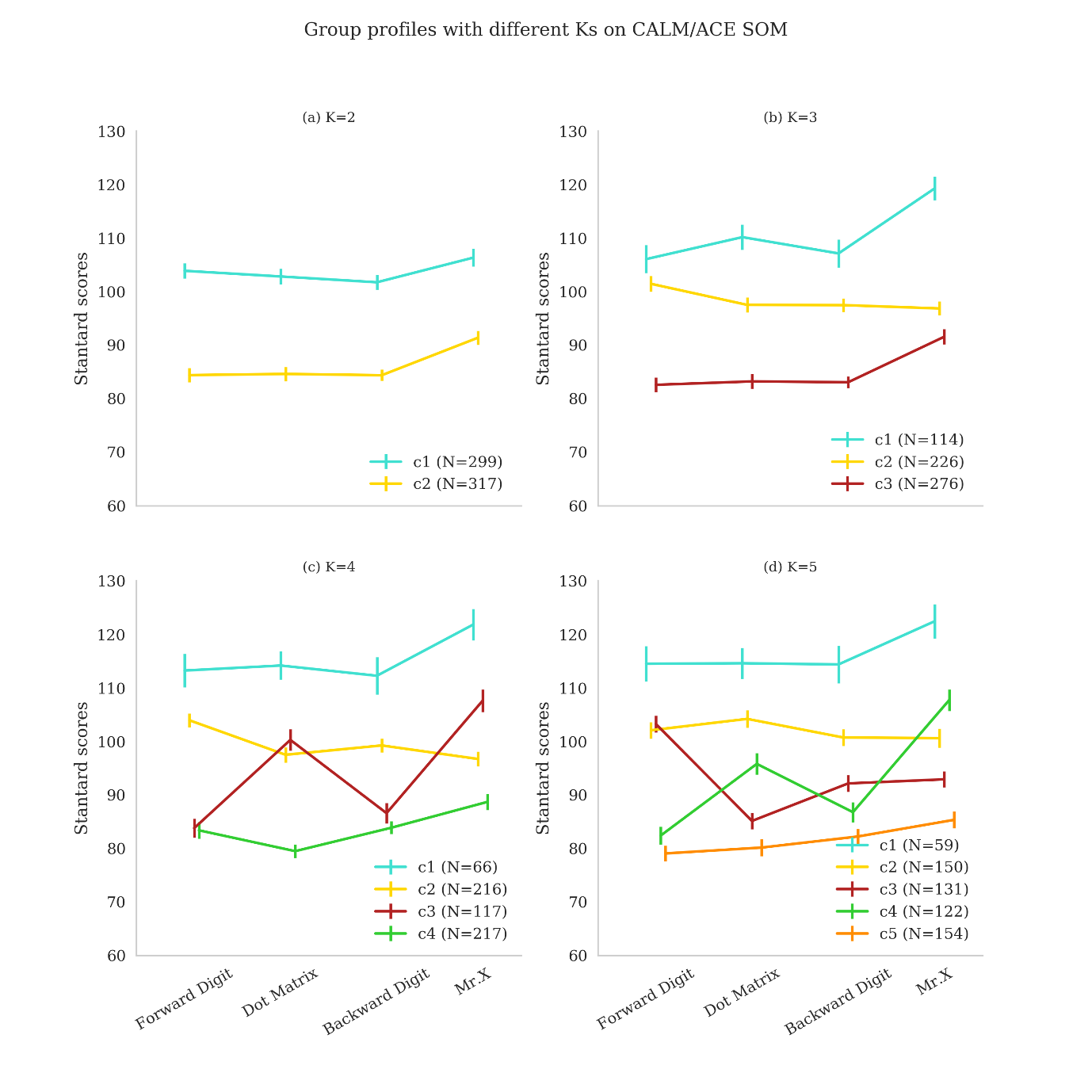
**

**Figure S5. Task performance profiles for a K of 4 on SOMs fit to the Pre- and Post-Training datasets respectively:**


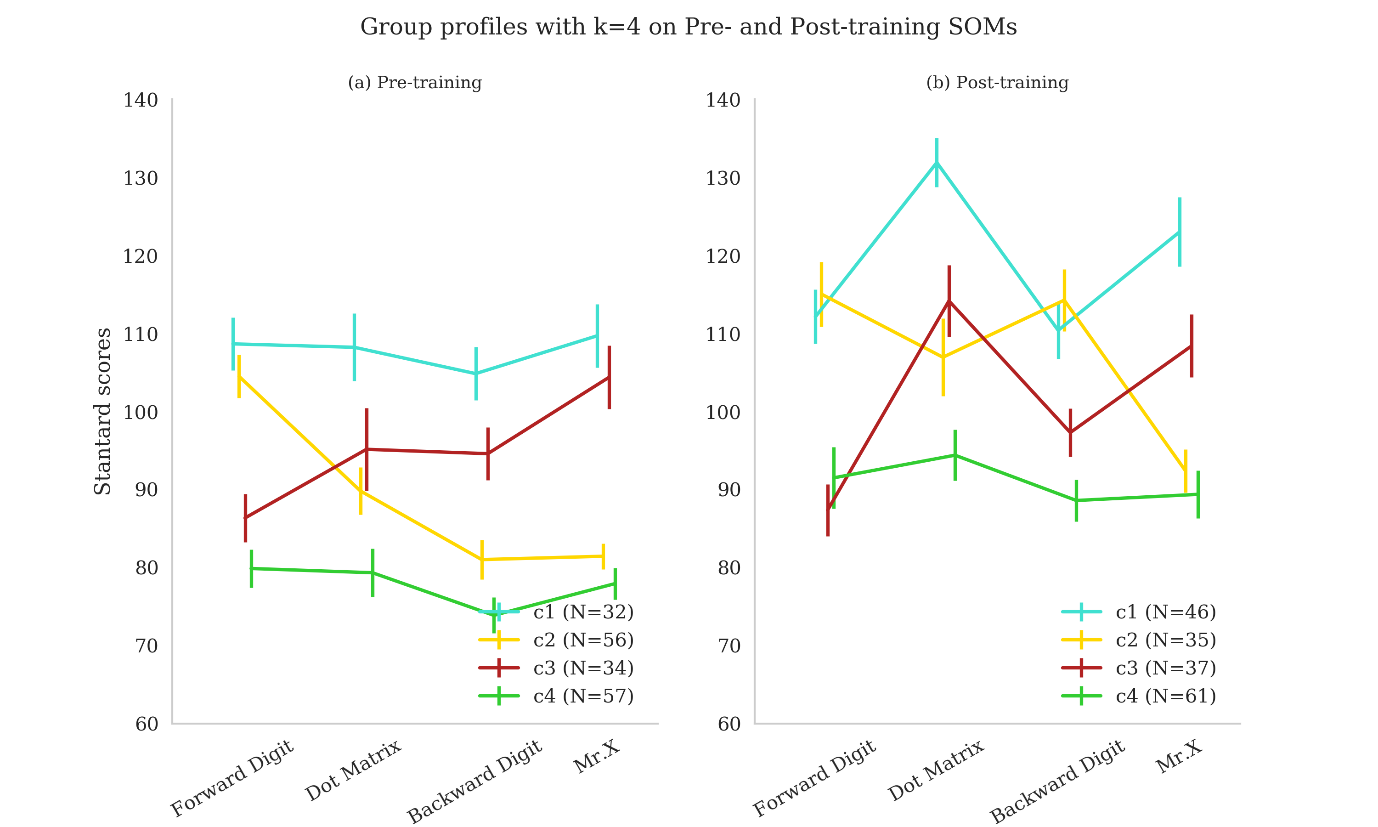


**SOM can detect change in task relationships present in the raw data**

In this section we highlight the unique contribution of SOM as a representative and noise-tolerant model of the original data. Specifically, the noise-reducing property of the learning algorithm lends itself to strengthening the relationships and the changes thereof that are present in the raw data (Yin, 2008). Using the model, two pairwise relationship changes were identified (Figure S6a and also see Figure 3 in main text), whereas only one (Backward digit-Mr. X) was found by computing raw cross-correlations, but with attenuated magnitude (Figure S6b).

**Figure S6. Between task correlations at Pre-Training, Post-Training, and the difference between the two; a comparison between SOM weights (a) and raw data (b). * denotes statistical significance at level of .05.**

**(a)**


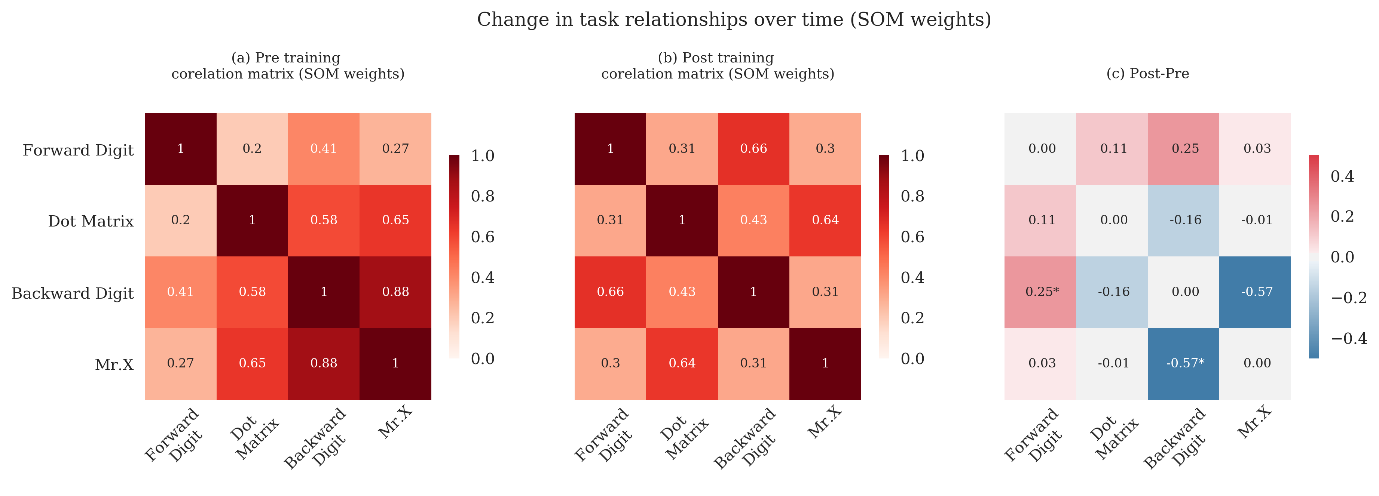


**(b)**


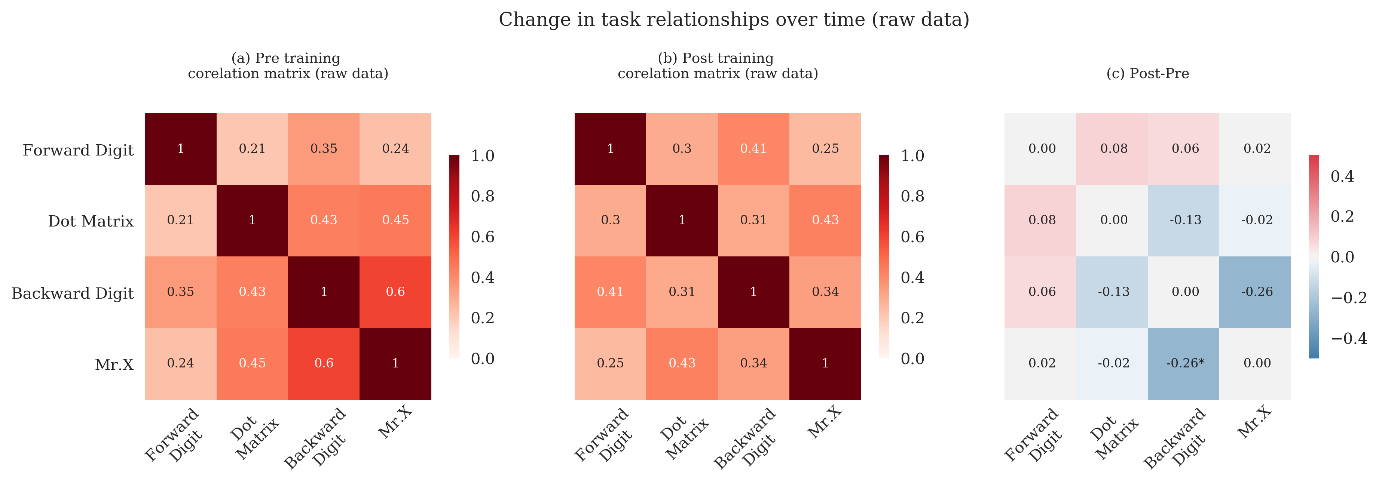


**Latent Change Score Modelling**

As another multivariate statistical approach, Latent Change Score Modelling (LCSM) has also been adopted to model changes over time in the cognitive training research literature (Karbach et al., 2017; Schmiedek et al. 2010). To characterize the extent to which LCSMs can be used to understand training-induced effects, we fit a multiple indicator univariate latent change score model to the combined training dataset using R’s Lavaan package and codes adapted from Kievit et al, 2017. We specified a model where all 4 cognitive measures load onto one latent variable “COG” (Figure S7).

As discussed in the main text, LCSMs assume measurement invariance between the time points of assessments, namely, the latent variables are constrained to have the same unstandardized factor loadings and intercepts over time. We tested the assumption by comparing different levels of invariance (i.e. configural, metric, scalar and strict) using a chi-square difference test (Widaman et al. 2010). The model failed to achieve metric measurement invariance (i.e. fixed factor loadings across time) when compared to a model that assumed configural measurement invariance: ∆χ^2^(3) = 8.125, *p* = 0.04, suggesting that the relationships between the observed and the latent variable at Pre- and Post- training are not equivalent. To identify the same latent construct longitudinally, metric or strict invariance must hold across times of measurement; configural and weak invariance are insufficient (Widaman et al. 2010). Therefore, the result raised questions about the suitability of LCSMs within the scope of the combined training study and necessitates the importance of having statistical alternatives that are also multivariate in nature.

**Figure S7. Latent Change Score Model on pre- and post-training data. COG_T1: latent factor at pre-training. COG_T2: latent factor at post-training. DIF: latent change score. There is no significant correlation between T1 and latent change (i.e. baseline score does not predict magnitude of improvement). Standardised parameters are displayed.**


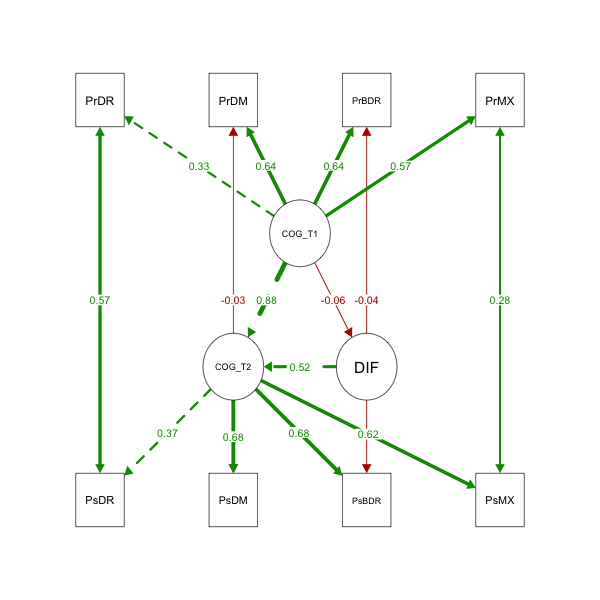


**Analysis of COGITO data**

To explore the scope of application of the SOM, an analysis using the same pipeline described in the main text was also applied to a separate dataset, namely the COGITO study from Schmiedek et al. (2010). COGITO data consisted of 204 participants who have completed an average of 100 hours of extensive training on working memory, processing speed and episodic memory (see the original published work for more details on training procedures and outcome measures). Six WM transfer measures including 3 updating and 3 complex span tasks were included in the initial training of SOM models for pre and post- assessments, respectively. Individual scores were shown in Figure S8a, clearly revealing a ceiling effect on most tasks with the exception of Spatial updating. Similarly, it can be observed that on tasks such as N-back and Counting span, weight values did not vary much across model nodes in both times of assessment. This means that the performance on these tasks were invariant to the differential profiles that might exist on the other tasks. A contrasting example would be the result shown in Figure 2a, where relatively clear gradients of SOM weights for the CALM/ACE data existed. Indeed, the authors of COGITO study also discussed the existence of ceiling effect and its potential implication for data analysis.

Therefore, we felt that these ceiling effects would constrains our ability to identify changes in task relationships or individual differences in training profiles and so decided against an in depth analysis of the data, or its inclusion in the main body of the text.

**Figure S8. Overview on SOM analysis result of COGITO data (Schmiedek et al, 2010). (a) Task performance at pre- and post-training across 6 WM transfer tasks included the reported training study. Theoretical upper limit is at 1. (b) Distribution of participants in each node of a SOM fitted to pre-training data. (c) Distribution of participants at time of post-training in each node of the same SOM as in S8b. Note that large proportion of participant aggregated to the bottom left region which correspond to ceiling performance across all measures.**

**
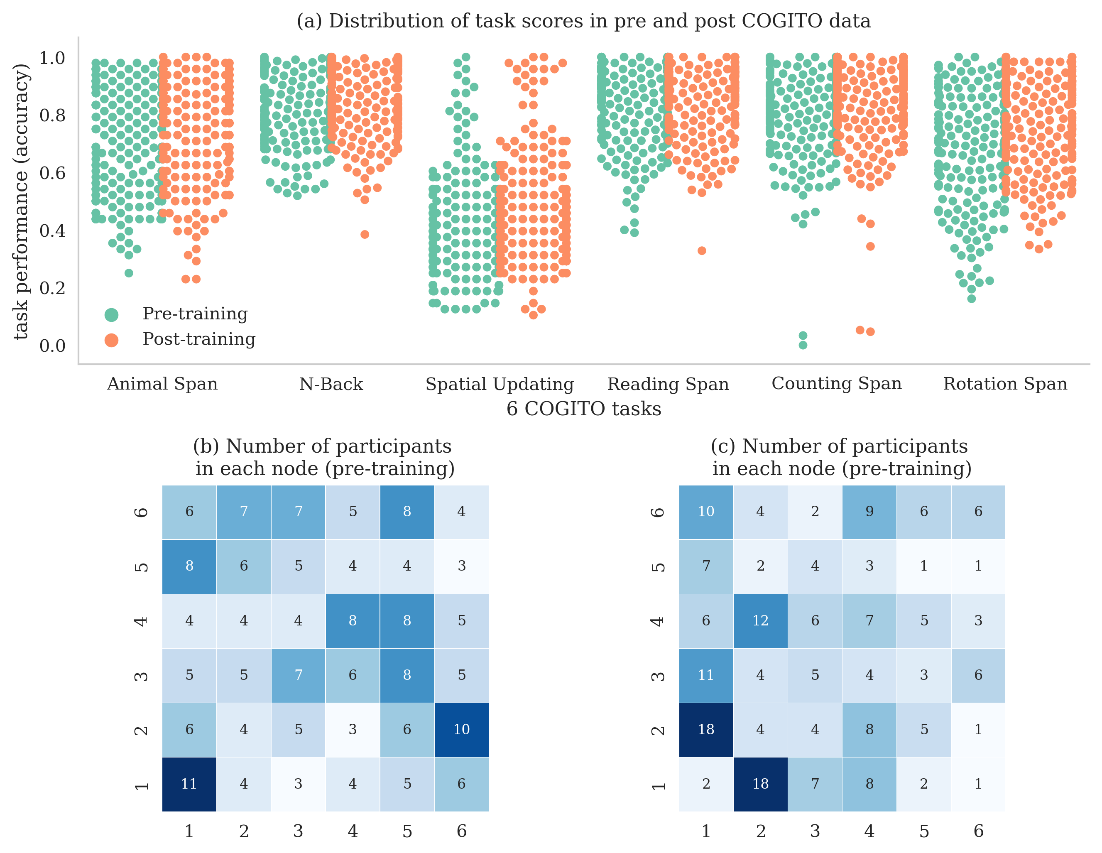
**

**Figure S9. Overview of SOM model weights trained on the COGITO (a) pre-training and (b) post-training data. It can be observed that on tasks such as N-back and Counting span (or even Animal span and Reading span), weight values did not vary much across model nodes in both times of assessment.**


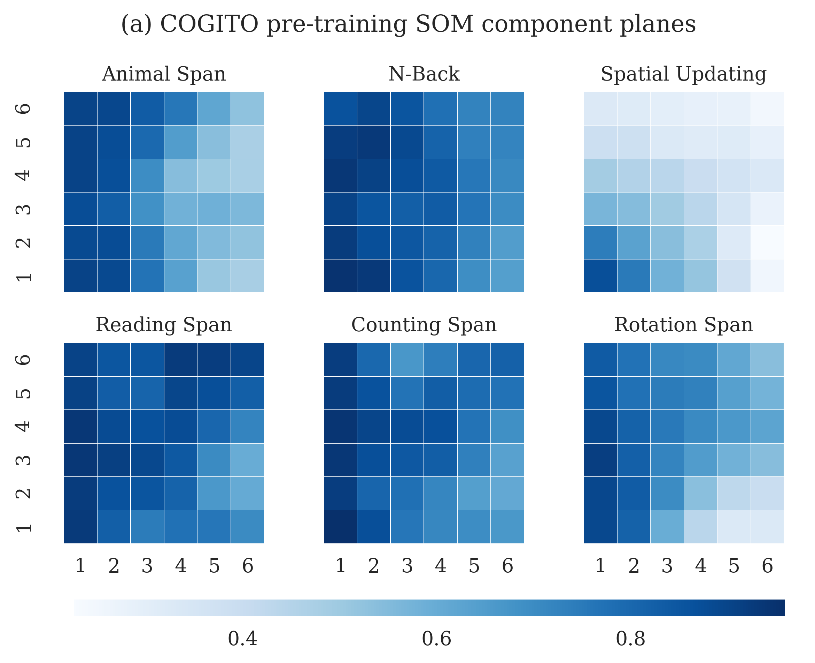

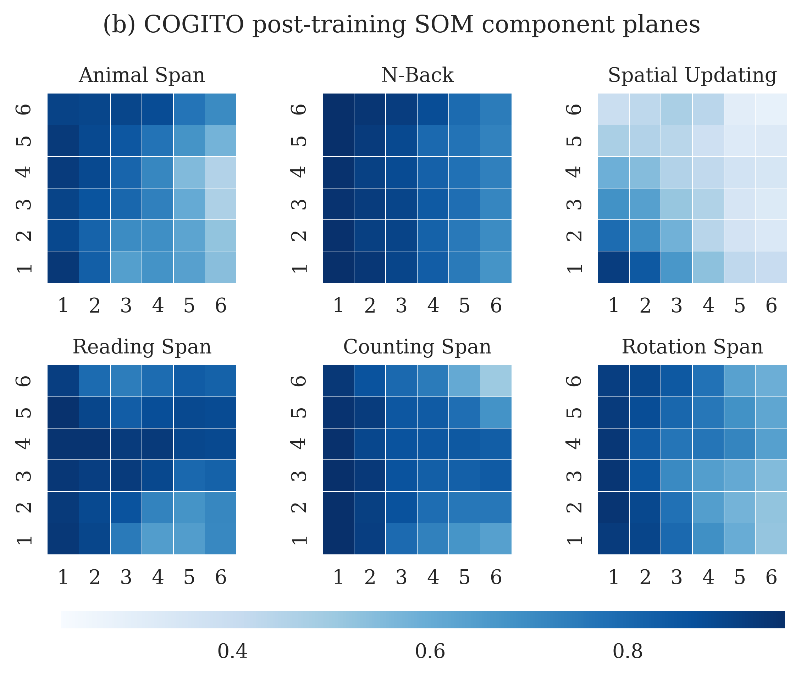


**Comparison with the control group**

Although the focus of the paper is not on the efficacy of the training, we have included below a brief-surface level summary of the control data analysis for those interested. The SOM proved capable of predicting all four variables in the control dataset above chance. Furthermore, these predictions did not become significantly worse for any of the four variables (Unlike Dot Matrix in the training data), indicating that their relationships were represented somewhere in the model fit to the CALM/ACE data at both pre and post training (Table S1.).

Interestingly, the correlational analysis of task relationships as represented by a SOM model fit to the pre-training control data and the post-training control data respectively showed that some of these task relationships also change over time (Figure S10), presumably due to practice effects/non-adaptive training effects. Specifically, the relationships between the Dot Matrix task and Forward Digit, Backward Digit, and Mr X were all altered significantly in the control groups data at post-training. Another candidate could be the regression to the mean, as most of these children were screened for some format of low cognitive impairment, poor performers with relatively intact WM have been over represented and are more likely to regress upwards to their mean. Note that these changes are not in line with the adaptive training group, which speaks to the fact that the correlational trajectory of these tasks over time is likely, non-linear.

In a similar vein, the clustering analysis below, that involved assigning control participants to one of the four groups identified with K-means clustering on the CALM/ACE Model (Figure S11.) shows that even non-adaptive training can result in substantial gains, leading in this case to a re-assignment of sub-group. Again, these are subject to individual differences.

| ***Table S1.*** CALM/ACE-SOM prediction errors for the Pre- and Post-training control samples, and direct comparison of these prediction errors relative to one another. Prediction error was defined as mean absolute difference between the predicted scores and true scores. *P-*values were derived from comparing the prediction errors against the corresponding chance level distributions. The chance levels were achieved by randomly shuffling the order of the predicted scores, then subtracting the true scores for 100 times within each cross-validation literation, to obtain a null distributions of mean absolute difference. | | | | | |
| --- | --- | --- | --- | --- | --- |
|  |  | **Forward Digit** | **Dot Matrix** | **Backward Digit** | **Mr. X** |
| **Pre-training**  **Controls** | **Prediction error (standard score)** | 11.35 | 13.07 | 10.57 | 13.73 |
|  | ***p*** | < .001*** | < .001*** | < .001*** | < .01** |
| **Post-training**  **Controls** | **Prediction error (standard score)** | 11.99 | 13.66 | 9.03 | 13.22 |
|  | ***p*** | < .001*** | < .001*** | < .001*** | < .001*** |
| **Pre vs Post Comparison Controls** | **Difference in Prediction error** | 0.64 | 0.59 | -1.54 | -0.15 |
|  | ***p*** | p=0.31 | p=0.34 | p=0.87 | p=0.63 |

Note. Asterisks denote statistical significance at *p< .05, **p < .01 or ***p< .00.

**Figure S10. Between task correlations at Pre-Training-Control, Post-Training-Control, and the difference between the two; a comparison between SOM weights (a) and raw data (b). * denotes statistical significance at level of .05.**

**
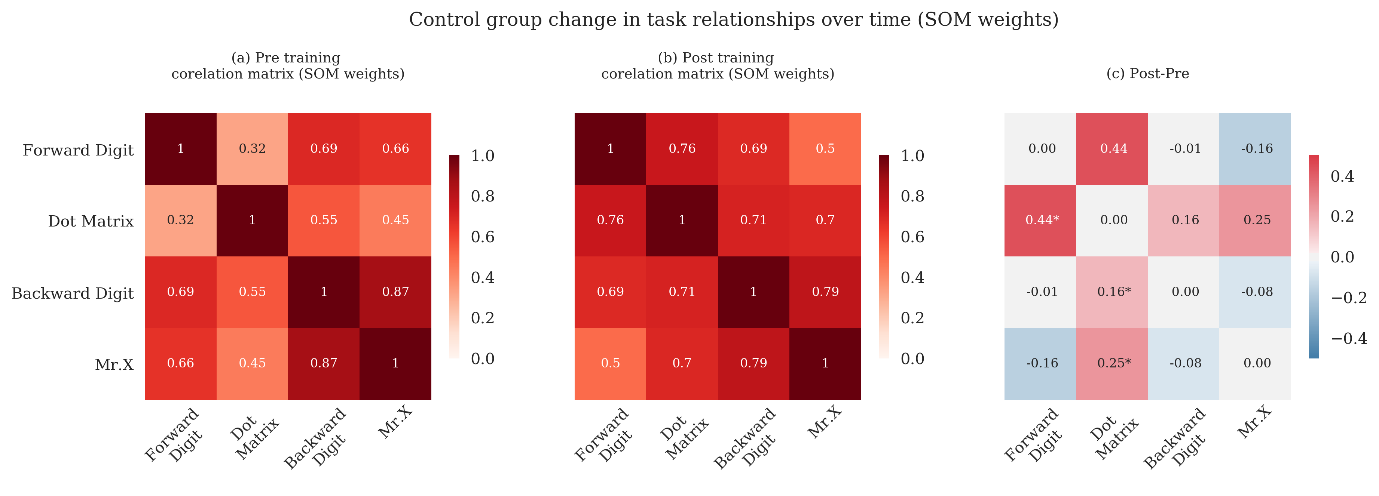

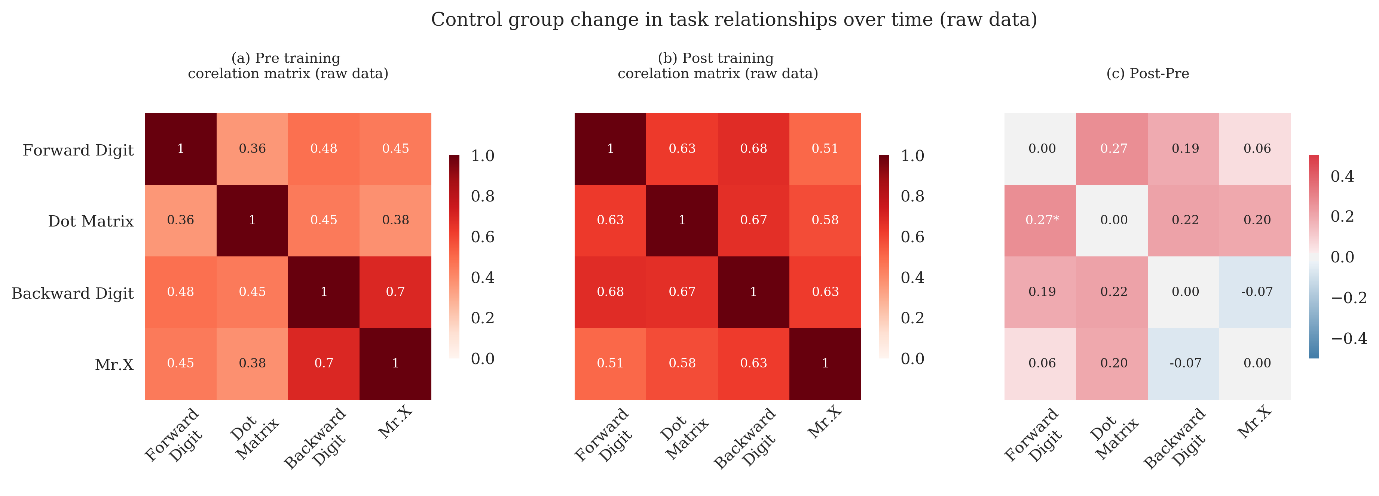
**

**Figure S11. Results of K-mean clustering and comparison of subgroup profiles in controls.** SOM nodes were partitioned into 4 clusters. (a). Pre-Training-Control task performance profiles. (b). Post-Training-Control task performance profiles (c). Improvement-Profiles. Error bar indicates 95% confidence interval.


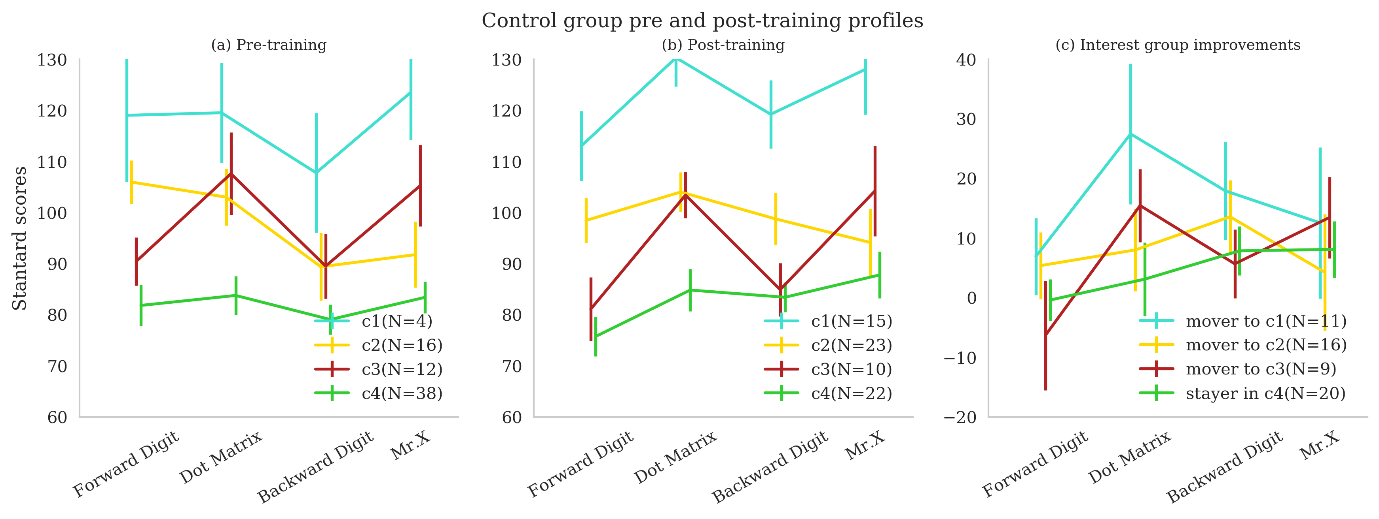


**References**

Widaman, K. F., Ferrer, E. and Conger, R. D. (2010), Factorial Invariance Within Longitudinal Structural Equation Models: Measuring the Same Construct Across Time. Child Development Perspectives, 4: 10-18. doi:[10.1111/j.1750-8606.2009.00110.x](https://doi.org/10.1111/j.1750-8606.2009.00110.x)

Kievit, R. A., Brandmaier, A. M., Ziegler, G., van Harmelen, A. L., de Mooij, S. M., Moutoussis, M., ... & Lindenberger, U. (2017). Developmental cognitive neuroscience using Latent Change Score models: A tutorial and applications. *Developmental cognitive neuroscience*.

Yin, H. (2008). The self-organizing maps: background, theories, extensions and applications. In Computational intelligence: A compendium (pp. 715-762). Springer, Berlin, Heidelberg.
